# KnowYourCG: Facilitating base-level sparse methylome interpretation

**DOI:** 10.1126/sciadv.adw3027

**Published:** 2025-10-24

**Authors:** David C. Goldberg, Hongxiang Fu, Daniel Atkins, Ethan Moyer, Chin Nien Lee, Yanxiang Deng, Wanding Zhou

**Affiliations:** ^1^ Center for Computational and Genomic Medicine, The Children’s Hospital of Philadelphia, PA 19104, USA.; ^2^ Department of Pathology and Laboratory Medicine, University of Pennsylvania, Philadelphia, PA 19104, USA.

## Abstract

Decoding DNA methylomes for biological insights is critical in epigenetics research. We present KnowYourCG (KYCG), a data interpretation framework designed for functional DNA methylation analysis. Unlike existing tools that target genes or genomic intervals, KYCG features direct base-level screenings of diverse biological and technical influences, including sequence motifs, transcription factor binding, histone modifications, replication timing, cell-type–specific methylation, and trait associations. Through implementing efficient infrastructure that rapidly screens and investigates thousands of knowledgebases, KYCG addresses the challenges of data sparsity in various methylation datasets, including low-pass or single-cell DNA methylomes, 5-hydroxymethylation (5hmC) profiles, spatial DNA methylation maps, and array-based datasets for epigenome-wide association studies. Applying KYCG to these datasets provides valuable insights into cell differentiation, cancer origins, epigenome-trait associations, and technical issues such as array artifacts, single-cell batch effects, and Nanopore 5hmC detection accuracy. Our tool simplifies large-scale methylation analysis and integrates seamlessly with standard assay technologies.

## Teaser

KnowYourCG is a scalable, CpG-based framework for DNA methylome interpretation for broad biological and technical links.

## INTRODUCTION

Modified cytosine 5′-carbon at the CpG dinucleotide context is one of the most studied epigenetic marks in higher eukaryotes. In mammals, DNA methylation extensively implicates gene regulation, genome evolution, organismal development, and disease (*1*). Despite the prevalent interest in characterizing the DNA methylome, understanding the functional implications of methylation changes can be difficult. This is partly because DNA methylation is encoded on specific sequence units, e.g., CpG dinucleotides, but is also highly plastic and jointly governed by multiple intrinsic and external factors, such as cell identity (*2*), genetics (*3*), pathology (*4*), sex (*5*), age (*6*), and other environmental conditions (*7*). Functional DNA methylation analysis often demands awareness of the sequence structures and all explicit and hidden biological covariates (*8*) and technical confounders (*9*).

Effective computational methods for mining biological links from DNA methylation data have been lacking compared to their gene expression counterparts (*10*–*12*). Most functional enrichment analysis methods for DNA methylation data piggyback on tools initially designed to investigate gene sets [e.g., DAVID (*12*)] and genomic intervals [e.g., HOMER (*13*) and GREAT (*14*)]. Methods specifically designed for DNA methylation data follow a similar gene-centric (*15*, *16*) or genomic interval–based approach (*14*, *17*, *18*). In other words, investigators must first link CpGs to genes or form a differentially methylated region (DMR) based on genomic proximity (*14*, *17*, *19*, *20*).

**Fig. 1.**
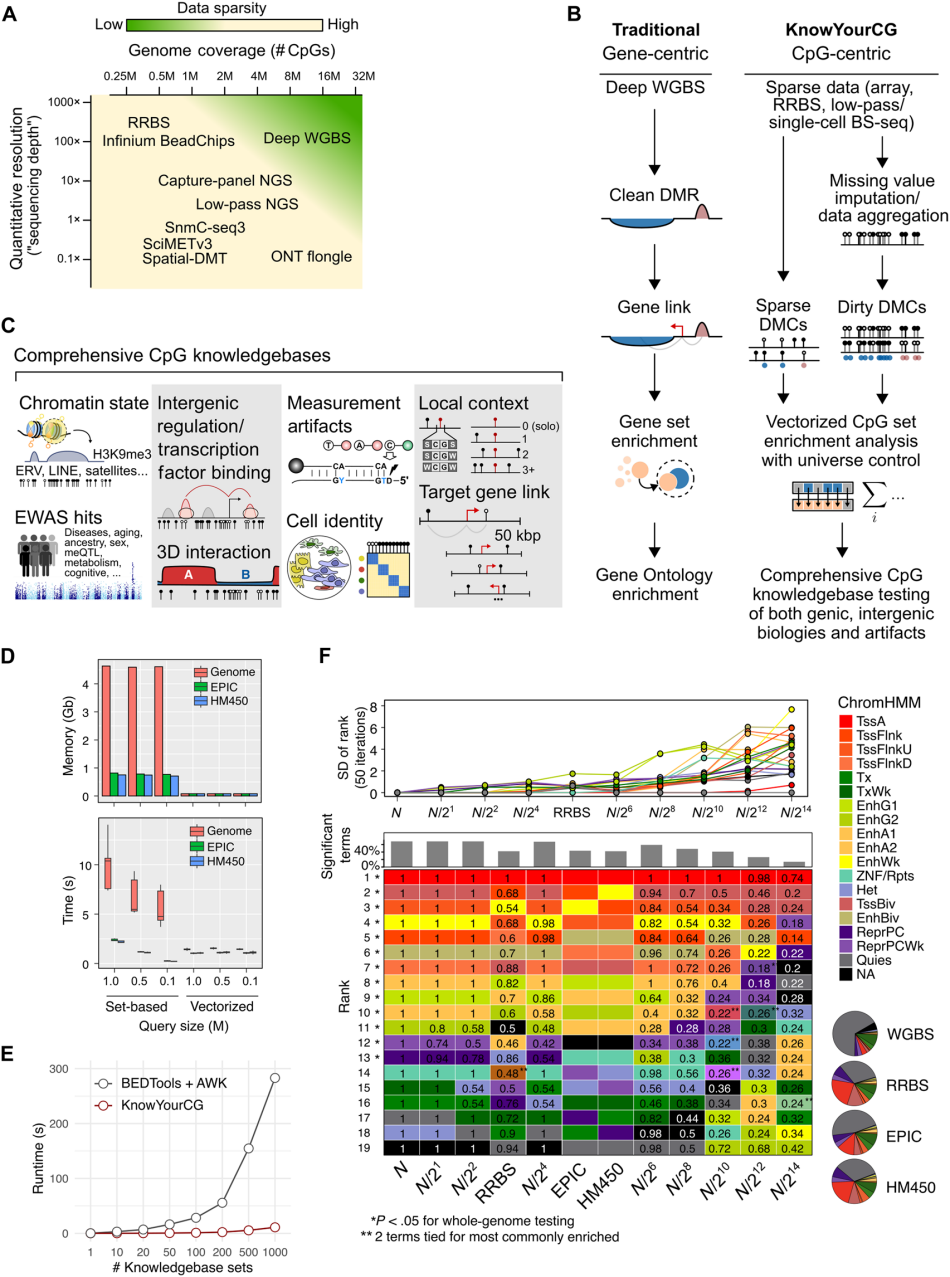
Overview of the KnowYourCG analysis framework. (**A**) Visualization of DNA methylation data sparsity in terms of the genome coverage and sequencing depth across common profiling methods. M, million; NGS, next-generation sequencing. (**B**) Schematic comparison between traditional gene-centric and KnowYourCG (KYCG) CpG-centric analytical workflows. BS-seq, bisulfite sequencing. (**C**) Overview of curated CpG knowledgebases used in KYCG for enrichment analysis. LINE, long interspersed nuclear element; kbp, kilo–base pair; ERV, endogenous retroviruses. (**D**) Memory and speed performance benchmarking of KYCG’s vectorized approach versus traditional set-based CpG representations. Gb, gigabytes. (**E**) Speed benchmarking of KYCG compared to a standard pipeline for computing enrichment statistics over increasing knowledgebase numbers. (**F**) Evaluation of enrichment testing in sparse datasets. ChromHMM state rankings were tested at varying levels of CpG sparsity from *N* (~28 million CpGs) to *N*/2^14^ (~1700 CpGs). *P* values are based on Fisher’s exact tests.

There are fundamental drawbacks to these strategies. First, DNA methylation data are inherently sparse due to CpG depletion outside CpG islands and additional sparsity introduced by practical constraints of profiling methods (Fig. 1A). The Infinium arrays, widely used in epigenome-wide association studies (EWAS), cover only 1 to 3% of the genomic CpGs (*9*). Reduced representation bisulfite sequencing (RRBS) covers ~10% but is limited to CpG-dense regions. Whole-genome bisulfite sequencing (WGBS) covers the entire genome but frequently lacks per-base depth and quantification granularity. Epitomizing both forms of sparsities, single-cell methylomes typically cover 1 to 10% of the entire CpG set in the genome (Fig. 1A) (*21*). These data sparsities make accurate definitions of DMRs difficult and often subjective, even when true differences exist.

Second, gene-centric approaches face challenges in establishing meaningful CpG-gene associations and unbiased gene weighting (*21*–*23*). Methylation at different gene regions plays distinct regulatory roles (*24*), and gene-centric analysis often misses biology at intergenic, geneless regions. Intergenic methylation is known to implicate cell replication (*25*, *26*), genome instability (*25*–*28*), cell differentiation (*2*, *29*), and aberrant writer/eraser enzyme activity (*30*, *31*). Because of the discrete nature of CpG dinucleotides and their depletion from deamination, proximity-based CpG-gene associations or DMRs may fail to reveal clear enrichment patterns. Instead, focal and dispersed methylation changes are more common and implicate transcription factor (TF) binding (*29*).

The alternative strategy to study functional links in DNA methylation data is to use CpGs as the units of analysis based on a fixed CpG index, as implemented in methods such as eFORGE (*32*, *33*), which were designed for array-based datasets with 20,000 to 900,000 probes (*34*). However, as newer datasets scale to whole-genome coverage (20 million to 30 million CpGs), overlap counting across hundreds to thousands of knowledgebase sets becomes computationally inefficient.

To address the above needs, we developed a comprehensive computational framework for DNA methylation data interpretation (Fig. 1B). KnowYourCG (KYCG) analyzes CpG sets for biological links and technical confounders. Capitalizing on a key technical innovation that rapidly enumerates CpG set differences across the whole genome, we achieve fast enrichment testing of methylomes against up to thousands of curated biological and technical covariates. Next, we first describe the implementation, after which we apply the tool to five broad application scenarios: (i) low-input DNA methylation profiles, including single-cell and spatial DNA methylation; (ii) 5-hydroxymethylation (5hmC) profiles and Nanopore-based direct detection; (iii) cell-type composition dynamics; (iv) interpretation of predictive machine learning tools such as epigenetic clocks and cancer classifiers; and last, (v) the detection of technical confounders. Collectively, we show that KYCG unveils interesting unreported links between CpG groups and demonstrated a variety of practical functionalities for analyzing large-scale DNA methylome data. Our tool is compatible with sequencing-based data and array platforms and has a user-friendly web-based application.

## RESULTS

### CpG-centric interpretation of sparse DNA methylomes

KYCG is a framework consisting of a web application, an R/Bioconductor application programming interface, a C command-line tool, and a database designed for DNA methylation data exploratory enrichment analysis, analogous to gene set enrichment analysis but focused on CpGs (Fig. 1B and fig. S1A). A CpG set linked to known biological functions, such as the specific binding sites of TFs, is called a knowledgebase set to distinguish it from the query. The significance of overlap between query CpGs and knowledgebase sets is evaluated using the hypergeometric distribution (Materials and Methods). To automate discovery, we uniformly processed 12,114,567 CpG-indexed knowledgebases for download and online query (Data and materials availability). These sets are constructed from human and mouse genome sequences, annotations, and public sequencing and array-based profiling (11,806 bulk and 480,012 single cells) and 1067 EWAS studies (Fig. 1C, table S1A, fig. S1B, and Materials and Methods).

To manage statistical complexity and improve interpretability, we grouped the CpG sets into biologically distinct testing knowledgebase domains representing separate hypothesis spaces with varying term counts, biological relevance, and structural organization. These domains are further classified into the following four major categories: (i) sequence features (e.g., *k*-mer, tetranucleotide, and transcription binding motifs), (ii) genomic features (e.g., chromatin states, histone modifications, gene links, transposable elements, TF bindings, and evolutionary conservation), (iii) trait associates (e.g., cell-type–specific methylations, human EWAS associates, and epigenetic clocks), and (iv) technical associates (e.g., sequence maskers, array hybridization, and extension masks). We extensively validated these knowledgebases, which form biologically relevant communities (fig. S1, C to E, and Materials and Methods). These testing domains define independent hypothesis spaces. Testing within domains preserves statistical power and biological focus.

To optimize performance, we used adaptive encoding to compress CpG sets, achieving compact disk storage and efficient in-memory manipulation (Materials and Methods). The comparison algorithm, implemented in C with bitwise vectorization, substantially accelerates the set overlap analysis. Our results demonstrate that for queries with 1 million CpGs, this method is ~10× faster and uses ~60× less memory than traditional set-based representations of CpGs. Unlike set representations, comparison time remains constant and scalable to large query sizes (Fig. 1D). Compared to a BEDTools-based pipeline of counting query overlaps (*35*), KYCG achieves a 25-fold speedup (Fig. 1E), supporting large-scale enrichment testing across thousands of knowledgebases. Similar performance gains extend to other functionalities, such as rapid methylation aggregation over knowledgebases (fig. S1F).

We first tested KYCG’s performance under query sparsity, as seen in RRBS, capture methylation sequencing (methyl-seq), and Infinium arrays, which target only a small subset of CpGs. To assess the enrichment testing feasibility, we simulated sparsity by downsampling CCCTC-binding factor (CTCF) binding–associated CpG sets from the full-genome set (*N* ~ 28 million) to *N*/2^14^. We then evaluated the stability of ChromHMM state rankings by comparing sparse and full-genome enrichment (Fig. 1F). Active promoters consistently ranked highest, but sparsity introduced variations. The top-ranking ChromHMM terms remained stable at sparsity levels down to *N*/2^10^ (~27,000 CpGs), with HM450, EPIC, and RRBS-based results resembling nonsparse predictions. However, the top enrichment term changed in 26% of runs at the extreme sparsity level (*N*/2^14^; ~1700 CpGs). These findings illustrated KYCG’s stability for enrichment testing with sparse CpG inputs.

### KYCG reveals biology from low-input, single-cell, and spatial DNA methylomes

**Fig. 2.**
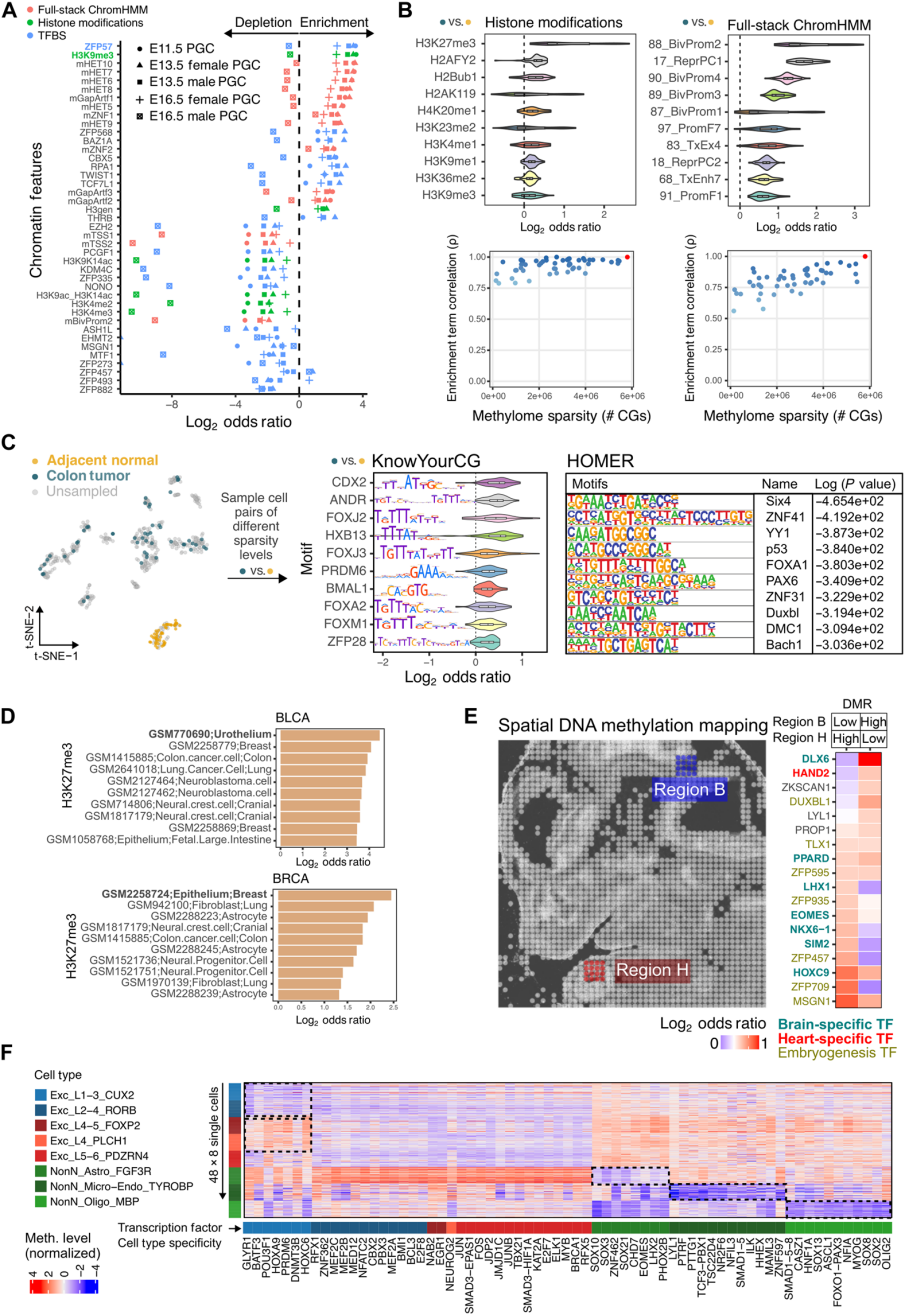
Application of KYCG to sparse low-input, single-cell, Nanopore, and spatial methylomes. (**A**) Enrichment analysis of sparse DNA methylomes (~2 million to 8 million CpGs) during PGC development. (**B**) Evaluation of 50 pairs of single-cell colon cancer versus adjacent normal methylomes. Spearman correlation of cancer hypermethylation enrichment results was tested relative to the least sparse pair (~6 million CpGs), indicated by the red dot. (**C**) t-SNE visualization of the selected 50 pairs of cells for comparison between the KYCG motif database and HOMER using single-cell colon cancer hypermethylation data. (**D**) Enrichment analysis of cell-type–specific H3K27me3 histone modifications of hypermethylated CpGs in bladder cancer (BLCA) and breast cancer (BRCA) TCGA datasets. (**E**) Neural tube and heart enrichment testing of TFBS from spatial mouse E11.5 embryo data. (**F**) Heatmap showing cell-specific TFBS methylation identified by aggregating methylation over KYCG knowledgebases. Forty-eight cells per major cell-type class are shown as rows, and TFBS knowledgebases are columns. Meth., methylation; t-SNE, t-distributed stochastic neighbor embedding; TCGA, The Cancer Genome Atlas.

Next, we evaluated KYCG’s performance in real sparse sequencing data by first analyzing methylomes (~2 million to 8 million CpGs) from various stages of primordial germ cell (PGC) development, where limited DNA precludes deep profiling (*36*). Enrichment analysis of methylated CpGs against TF binding sites (TFBS) and histone mark knowledgebases (Fig. 2A) showed that regions escaping global hypomethylation were enriched for heterochromatic (Het) marks, including histone H3 lysine 9 trimethylation (H3K9me3) and zinc finger protein 57 (ZFP57) binding. This enrichment was absent in male embryonic day 16.5 (E16.5) PGCs, consistent with known methylation rebound at this stage (*37*). These findings demonstrate KYCG’s ability to reveal biology at intergenic regions.

We next evaluated whether KYCG captures biology from highly sparse single-cell methylomes (200,000 to 1 million CpGs), a common scenario when pseudobulk aggregation is limited by biological availability or cost. In a pairwise comparison between a randomly selected single colon tumor cell and an adjacent normal cell, KYCG reveals the signature enrichment of hypermethylation at bivalent chromatin, marked by H3K27me3 and bound by Polycomb repressive complex members [e.g., polyhomeotic homolog 1 (PHC1), polycomb group ring finger 2 (PCGF2), jumonji and AT-rich interaction domain containing 2 (JARID2), ring finger protein 1 (RING1), enhancer of zeste homolog 2 (EZH2), etc.] (fig. S2A) (*38*). Hypomethylated CpGs were enriched in quiescent (Quies) and Het regions, Hi-C B compartments, and WCGWs (fig. S2B), as previously characterized (*25*). The result is robust to cell pairs of different sparsity levels. Notably, the cancer-specific hypermethylation pattern was robustly detected in extremely sparse methylome profiles covering as few as ~12,000 CpGs (~0.05% genomic CpGs), showing strong correlation with the most deeply sequenced cells (Fig. 2B). Similarly, KYCG also captured cell-type–specific differences, with differential methylation between single forkhead box protein p2–positive (Foxp2^+^) neurons and oligodendrocytes enriched at enhancer binding sites (fig. S2C) (*39*).

To assess KYCG’s advantage in sparse methylome analysis, we compared it to HOMER, a widely used genomic interval–based enrichment tool (*13*). We used the above colon cancer hypermethylation as our query and tested the enrichment of TF binding motifs. KYCG identified biologically relevant motifs, such as caudal type homeobox 2 (CDX2), a key player in intestinal differentiation and often acting as a tumor suppressor and a prognostic marker (*40*, *41*), as well as the FOX family and the androgen receptor ANDR, both implicated in colon cancer (*42*, *43*). Testing the larger DMRs against similar TF binding databases (Materials and Methods), HOMER missed the colon relevance and picked up general TFs, affecting cellular differentiation and proliferation instead, such as sine oculis homeobox 4 (SIX4) and zinc finger protein 41 (ZNF41) (Fig. 2C) (*44*, *45*). Notably, when using aggregated pseudobulks, HOMER did detect CDX2 and FOX motif enrichment. However, this signal diminished with smaller cell numbers (fig. S2D), suggesting that DMR calling may dilute the signal in sparse settings.

Furthermore, we observed that cancer-associated hypermethylation patterns align with the cancer cell’s tissue of origin. For example, while hypermethylated CpGs in TCGA bladder cancers were broadly enriched for H3K27me3 across many cell types, the strongest enrichment was observed when comparing H3K27me3 marks in immortalized urothelium cells. Likewise, breast cancer hypermethylation is most enriched in the same mark profiled from MCF7 breast epithelium cells (Fig. 2D).

To demonstrate KYCG’s broad applicability, we applied KYCG to a spatial DNA methylation dataset from a mouse E11.5 embryo (Fig. 2E) (*46*). Methylation differences between cells from two spatial regions (B and H) located near the brain and heart areas on the light-field image were analyzed. Differential methylation was primarily linked to embryogenesis-specific TFs, including zinc finger proteins, which is consistent with the developmental stage (Fig. 2E). Region B hypomethylation was enriched for brain-specific TFs [(e.g., peroxisome proliferator-activated receptor delta (PPARD), LIM homeobox 1 (LHX1), eomesodermin (EOMES), NK6 homeobox 1 (NKX6-1), single-minded homolog 2 (SIM2)], while region H hypomethylation was enriched for heart-specific factors such as heart and neural crest derivatives expressed 2 (Hand2) (*47*–*49*). Notably, the brain-specific TF distal-less homeobox 6 (DLX6) was hypomethylated in region H, suggesting a preference for methylated DNA binding. These results highlight KYCG’s capability to resolve region-specific methylation differences and connect them to biological processes.

Aggregating methylation signals can mitigate missingness in single-cell datasets. However, large bin– or continuous genomic interval–based aggregation may obscure biologically relevant trans-acting features that span multiple genomic sites. Using KYCG’s fast aggregation capability (fig. S1F), we analyzed 1188 TFBS knowledgebases across 4000 single cells from 20 brain cell types to uncover transcriptional networks underlying cell identity (*50*). Differential methylation analysis revealed distinct patterns (Fig. 2F), such as hypomethylation at oligodendrocyte transcription factor 2 (OLIG2), SRY-box transcription factor 2 (SOX2), and SRY-box transcription factor 8 (SOX8) binding in oligodendrocytes, key regulators of their development (*51*, *52*), and at nuclear factor, interleukin 3 regulated (NFIL3) and lymphoblastic leukemia derived sequence 1 (LYL1) binding in microglia, linked to immune function (*53*, *54*). In addition, TFBS methylation distinguished superficial cortical neurons (L1-3/L2-4) from deeper layers (L4-5/L5-6), highlighting epigenetic regulation of cortical layer development. These findings demonstrate KYCG’s utility for dimensionality reduction and feature aggregation in sparse single-cell data.

### KYCG facilitates 5hmC analysis and assesses Oxford Nanopore Technologies direct detection

5hmC, an intermediate in 5-methylcytosine (5mC) oxidation and demethylation, plays a critical role in epigenetic cell identity. Despite its importance, 5hmC exhibits dynamic and sparse distribution (*55*–*59*). Even in brain tissues, where 5hmC is most abundant, it reaches only 10 to 20% of 5mC levels (*60*), posing substantial challenges for data analysis (*21*, *61*).

**Fig. 3.**
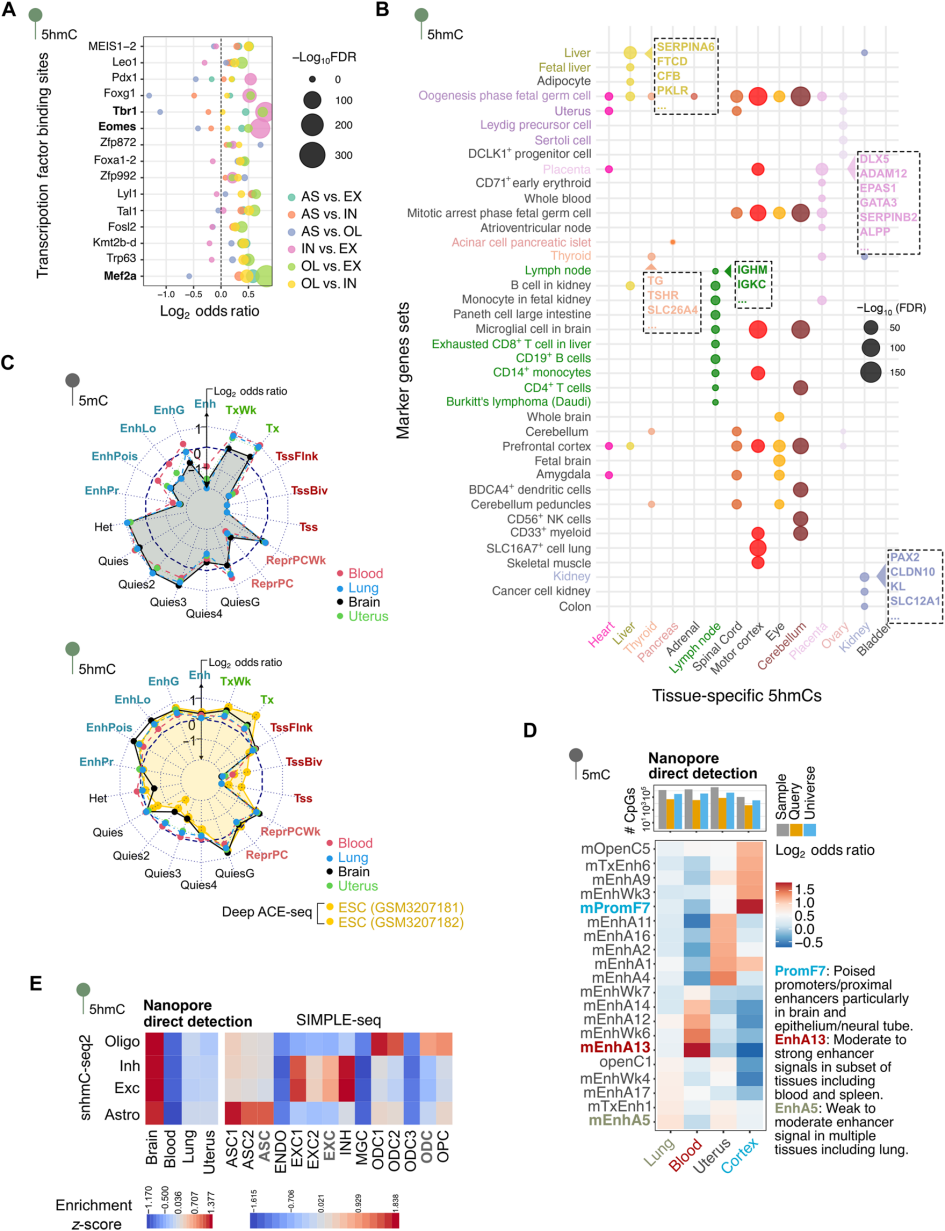
Application of KYCG to 5hmC analysis and ONT direct detection. (**A**) Pairwise comparison of 5hmC profiles in major brain cell types (astrocytes, oligodendrocytes, excitatory neurons, and inhibitory neurons) derived from snmC-seq2 data. AS, astrocytes; OL, oligodendrocytes; EX, excitatory; IN, inhibitory. (**B**) Marker gene enrichment for hyper-5hmC from human bisulfite APOBEC-coupled epigenetic sequencing (bACE)-array data. (**C**) ChromHMM state enrichment of ONT-derived 5mC and 5hmC signals across four mouse tissues (lung, blood, uterus, and cortex) and deep ACE sequencing (ACE-seq). (**D**) Tissue-specific chromatin state enrichment of tissue-specific 5mC from ONT. (**E**) Comparison of ONT-derived 5hmC profiles and single-cell 5hmC datasets (SIMPLE-seq and snmC-seq2). ASC, astrocytes; ODC, oligodendrocytes; OPC, oligodendrocyte precursor cell; Exc, excitatory; Inh, inhibitory.

To address the challenges of analyzing sparse 5hmC data, we tested KYCG on 5hmC profiles from recent single-cell studies. Using snhmC-seq2 data (*57*), we evaluated brain cell types where 5hmC was measured at only 0.2 to 1% CpGs in astrocytes and oligodendrocytes. Pairwise comparisons revealed that 5hmC differences between cell types were enriched in TF binding and genes linked to brain cell differentiation programs (Fig. 3A and fig. S3, A and B). T-box brain transcription factor 1 (TBR1) and Eomes emerged as the most significant TFs discriminating between excitatory and inhibitory neurons. The two TFs are essential for the development of glutamatergic excitatory neurons in the cerebral cortex and are typically absent in GABA-releasing inhibitory neurons (*62*, *63*). Besides, Myocyte Enhancer Factor 2A (Mef2a), an important transcription factor for excitatory neurons (*64*), emerged as a TF with binding significantly enriched at 5hmC differences between excitatory neurons from oligodendrocytes (Fig. 3A).

In nonbrain high-turnover tissues, 5hmC is even scarcer (*60*), as 5hmC is a poor substrate for DNA methyltransferase 1 (DNMT1) and unmaintained in rapidly dividing cells (*65*, *66*). This ultrasparsity leaves the interval and per-locus analysis of genome-wide 5hmC patterns largely impractical (*61*). To assess KYCG’s utility in this context, we analyzed 104 human 5hmC profiles across 25 tissue types generated using the bACE-array technology (*67*), applying KYCG to evaluate tissue-specific 5hmC signals (Fig. 3B). 5hmC sites in proliferative tissues, such as lymphocytes and placenta, were enriched near marker genes of corresponding cell types (Fig. 3B). For example, the placenta-specific gain of 5hmC is localized to *ADAM12* and *EPAS1*, genes expressed in trophoblasts that regulate placental vascularization, nutrient availability, and immune tolerance (*68*–*70*). In lymph nodes, 5hmC was enriched near *IGHM, IGKC*, and other genes involved in B cell signaling and antibody production (*71*, *72*). These observations demonstrate KYCG’s versatility in uncovering tissue-specific epigenetic regulation from ultrasparse 5hmC datasets.

Oxford Nanopore Technology (ONT) is an emerging approach to directly discriminate 5mC, 5hmC, and unmodified C from ion current signals (*73*, *74*), bypassing cytosine deamination methods that cannot separate 5mC and 5hmC (*75*). However, ONT’s 5hmC detection remains undercalibrated (*74*, *76*), and per-site accuracy is difficult to assess due to the sparse and heterogeneous nature of 5hmC. To address this, we used KYCG to evaluate the biological relevance of ONT-based 5mC and 5hmC signals across four mouse tissues (lung, blood, uterus, and cortex) profiled with low-pass Flongle flow cells (~1 million CpGs per sample).

Our results showed that ONT-derived 5mC and 5hmC maps are consistent with established biology. 5mC was enriched at gene bodies (Tx) and Het (Fig. 3C and fig. S3C) (*67*). From the sparse methylomes, we identified specific methylation patterns contrasting one sample against the others. These patterns exhibited tissue-specific chromatin state enrichment, such as PromF7 (*77*) in brain cells and EnhA13 (*77*) in blood and immune cells (Fig. 3D). 5hmC shares 5mCs’ enrichment in gene bodies but is depleted in Het. Furthermore, 5hmC was enriched at enhancers, where 5mC is depleted, highlighting the unique role of 5hmCs in ten-eleven translocation (TET)-mediated active demethylation and cis-regulation. The ONT 5hmC enrichment patterns closely mirrored deep ACE sequencing (ACE-seq) data, supporting its biological accuracy (Fig. 3C). Further validation using single-cell 5hmC datasets (SIMPLE-seq and snhmC-seq2) showed strong cross-dataset concordance. Comparing these with ONT 5hmC signals, all brain cell types showed higher enrichment in brain ONT profiles compared to blood, lung, and uterus (Fig. 3E). While limited by the bulk nature of the ONT data, these findings support the broad biological relevance of ONT in resolving 5hmC landscapes.

### KYCG detects cell composition dynamics through enrichment testing

**Fig. 4.**
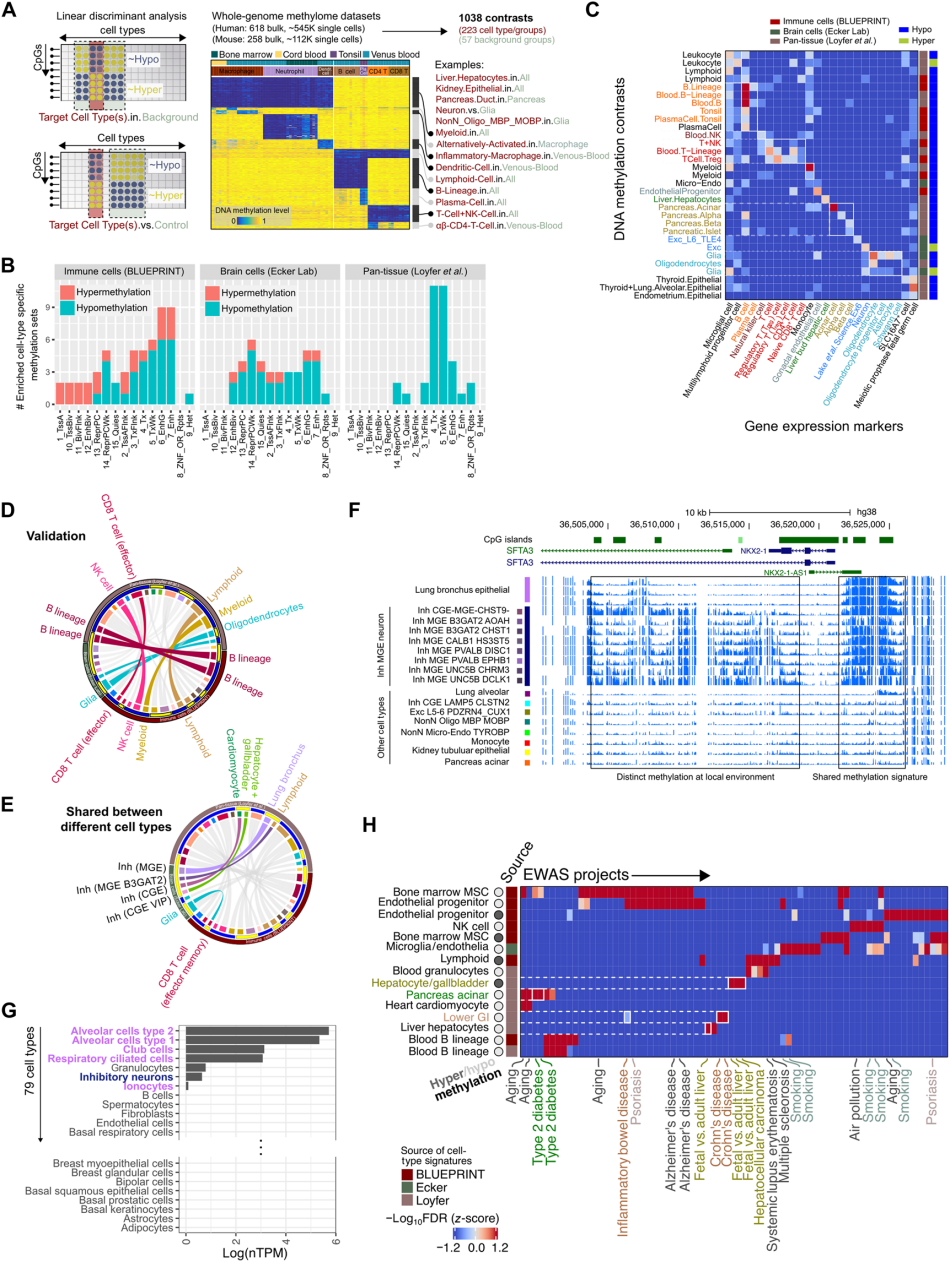
Detection of cell composition dynamics through KYCG cell-type–specific DNA methylation signature enrichment. (**A**) Construction of cell-type–specific methylation knowledgebases with contrasts defined as pairwise comparisons of cell-type groups. Dendr., dendritic; Pla., plasma. (**B**) Chromatin state enrichment of hyper– and hypo–cell-type–specific methylation knowledgebases for immune, brain, and pan-tissue datasets. (**C**) Methylation signatures of cell types are enriched in marker genes for the corresponding cell type. (**D**) Validation of cell-type–specific methylation knowledgebases across datasets using normalized pointwise mutual information (NPMI). (**E**) Shared methylation signatures between unrelated cell types. CGE VIP, caudal ganglionic eminence vasoactive intestinal peptide–expressing interneurons. (**F**) Comparison of local methylation environment analysis at the *NKX2-1* locus for inhibitory neurons and lung bronchus cells with other cell types. (**G**) Expression analysis of *NKX2-1* across 79 cell types. nTPM, normalized transcripts per million. (**H**) Heatmap showing enrichment of EWAS hit CpG sets in cell-specific methylation CpG sets. The –log_10_ FDR values from the enrichment tests are *z*-score normalized within each trait (columns). Trait-related methylation enriches the cell types where the trait manifests. MSC, mesenchymal stem cell; GI, gastrointestinal.

DNA methylation has long been established as a robust biomarker to discriminate cell types and analyze their composition in heterogeneous tissues (*78*). We reason that enriching methylation changes in cell-type–specific methylations would inform cell composition dynamics. To test this, we compiled KYCG knowledgebases, each holding CpG sites whose methylations discriminate two cell-type groups (a cell type contrast), including commonly used “one-versus-rest” comparisons (Fig. 4A and Materials and Methods). We used a nonparametric linear discriminant analysis approach to construct these knowledgebases while prioritizing CpGs showing large methylation differences between the contrasting groups (Fig. 4A).

To verify the quality of cell-type–specific methylation sets, we investigated their genomic distribution and validated sets across studies. First, consistent with prior reports (*79*, *80*), cell-type–identifying methylation signals were more often based on the absence than the presence of methylation in the target cell types (Fig. 4B) and represent cell-type–specific enhancer chromatin (fig. S4A) (*81*). Second, cell-type–specific methylations likely regulate marker genes of the target cell type, suggesting an immediate transcriptional consequence (Fig. 4C). Genomic proximity analysis found that hypermethylation knowledgebases are more spatially clustered than the hypomethylation sets, suggesting their localization to CpG islands and involvement with the target gene expression (fig. S4B). Third, using normalized pointwise mutual information (NPMI) to measure set overlaps, we found that related cell types from different sequencing projects were associated with similar methylation signatures with concordant directionalities (Fig. 4D). Last, the cell-type–specific methylations are linked to cell lineage specification. For instance, brain cell methylation signatures are enriched in genes implicated in neurodevelopment and the differentiation of the specific neuron or glial cell types (fig. S4C).

Some unrelated cell types share methylation changes at overlapping CpG sites, suggesting regulatory network reuse (Fig. 4E). For example, inhibitory medial ganglionic eminence (MGE) neurons and lung bronchus cells, despite functioning in disparate organ systems and arising from different developmental origins, shared methylation signatures (Fig. 4, E and F). Although unexpected, we confirmed that these regions are indeed similarly methylated at the *NKX2-1* locus and share similar *NKX2-1* expression patterns relative to all other cell types they were compared to (Fig. 4G).

Cell composition dynamics may be mechanisms of methylation associations in EWAS studies of bulk tissues. Using our cell-specific knowledgebases, we tested whether KYCG could detect cell composition changes across disease states. We observed a concordant enrichment of trait-associated CpGs in the corresponding cell-type signatures (Fig. 4H and table S1B). For example, inflammatory bowel disease and Crohn’s disease–associated CpGs were enriched in lower gastrointestinal cell markers, while CpGs with type 2 diabetes–linked methylation showed enrichment in pancreatic cells. Similarly, methylation variations interrogated in liver aging and hepatocellular carcinoma studies were enriched in CpGs carrying hepatocyte-specific methylations. These observations likely reflect disease-associated shifts in cell-type proportions or aberrant methylation at cell identity–linked sites.

### KYCG facilitates machine learning model interpretation

DNA methylation–based predictive models have been widely used in translational applications. However, interpreting these “black-box” models remains challenging. We hypothesize that KYCG could reveal the workings of predictive models by analyzing model features. Below, we focus on epigenetic clocks and cancer classifiers as two examples.

**Fig. 5.**
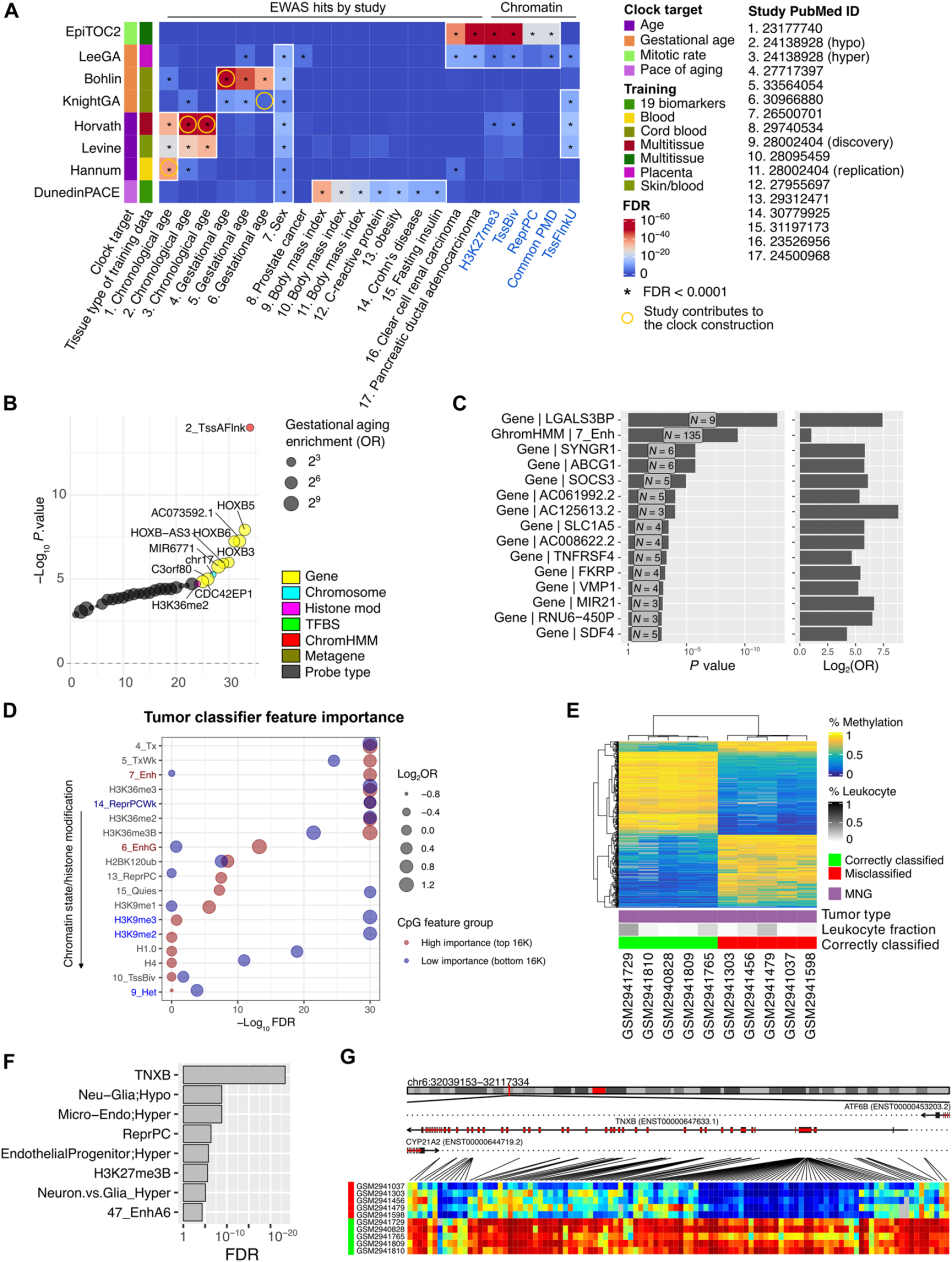
Machine learning model interpretation with KYCG. (**A**) Enrichment testing of eight epigenetic clocks reveals feature-specific associations with chromatin states and 17 EWAS studies. *P* values are based on Fisher’s exact tests before FDR correction. (**B**) Bohlin gestational age clock features enrichment in *HOXB* gene clusters and H3K36me2 histone modifications. OR, odds ratio. (**C**) Enrichment of DunedinPACE clock probes in genes and chromatin states. (**D**) Enrichment analysis of high- and low-importance CpG features from cancer classifiers. (**E**) Differential methylation enrichment analysis between correctly and incorrectly classified tumor samples. (**F**) Misclassified meningiomas compared to correctly classified tumors reveal CpG enrichments in neuronal, endothelial, and microglial signatures. *P* values are based on Fisher’s exact tests before FDR correction. (**G**) Heatmap of TNXB-associated methylation differences between correctly and incorrectly classified meningiomas. TNXB, tenascin XB.

We queried eight epigenetic clocks that predict chronological aging and biological causes that alter organismal aging. First, we observed that different clock models’ features are associated with different enrichment terms, potentially reflecting the clocks’ prediction targets (Fig. 5A). The DunedinPACE clock, designed to predict the pace of aging from 19 different physiological measures (*82*), was highly enriched in sites with methylations linked to body weight and metabolic traits. The EpiTOC clock measures mitotic activity (*83*) and was enriched in cancer studies, partially methylated domains (PMDs), and Polycomb group targets. The Horvath, Levine, and Hannum clocks that predict chronological or phenotypic age were enriched in aging EWAS studies from independent cohorts not seen during training by the respective clock. Bohlin and Knight gestational age clocks (*84*, *85*) were enriched in independent gestational age EWAS studies (*86*), while the Lee clock (*87*), trained on placental tissues, was also enriched in one gestational age study. Similar to EpiTOC, it was also enriched in cancer-associated methylations, bivalent chromatin, Polycomb group targets, and PMDs.

Besides linking the clock features to related traits, KYCG also generated hypotheses regarding the models’ workings. The Lee clock enrichment likely reflects placental tissue’s high proliferation and cancer-like properties and may explain the poor performance of other cord blood–trained clocks on placental samples (*87*). For the Horvath and the Hannum clocks that predict chronological ages, we observed enrichments in cell-specific methylations from immune cell types such as monocytes, natural killer (NK) cells, and dendritic cells (fig. S5A). These enrichments reflect altered blood composition during the aging process (*88*) and are leveraged by epigenetic clocks to predict age (*89*). Compared to other aging clocks, the Bohlin gestational clock was enriched in *HOXB* genes and histone H3K36me2 marks (Fig. 5B), suggesting that the clock might have used the methylation of homeobox (HOX) genes, which are important for gestational development and body patterning (*90*), and the methylation gain might be mediated by H3K36me2, which recruits DNMT3s via the PWWP domains (*91*). The same *HOXB3* site (cg15908709) can also be associated with gestational age in an independent dataset (fig. S5B) (*86*), validating this link. Last, KYCG found an enrichment of DunedinPACE clock features in overweight phenotypes, e.g., body mass index, obesity, and hepatic fat, as well as inflammatory disease signaling, e.g., Crohn’s disease, irritable bowel syndrome, and C-reactive protein (fig. S5C). Notably, DunedinPACE features are spatially linked to the gene *LGALS3BP* (Fig. 5C), which regulates immune responses in colon epithelial cells (*92*), cancer (*93*), HIV infection (*94*), and organ decline (*95*), suggesting a potential mechanism of the clock tracking diseases via the epigenetic regulation of a key circulating glycoprotein.

We next asked whether KYCG could help interpret cancer classifiers (*96*). We trained a random forest classifier on 2801 public brain tumor methylomes from more than 80 tumor classes (Materials and Methods). KYCG found that features with the highest importance scores were enriched in enhancers and actively transcribed genes, whereas less important CpGs were more enriched only in gene bodies (Fig. 5D). This highlights that the tumor cells of origin and the regulatory network underlying the cell identity difference are the main signal sources in cancer classification.

Furthermore, KYCG can help explain misclassifications. For example, we compared five correctly classified meningiomas to five misclassified tumors (Materials and Methods), separated by the leading principal component (Fig. 5E and fig. S5D). The 200 CpGs with the greatest positive loading scores along the leading principal component were enriched in neuronal, endothelial, and microglia signatures, suggesting that these samples may have different cells of origin (Fig. 5F). Linear modeling between the classification groups identified 30,686 differentially methylated CpGs that distinguished correct classification and misclassifications. These CpGs were enriched in *TNXB* (Fig. 5, F and G), which was previously shown to be differentially methylated across the dura and leptomeningeal layers of the meninges (*97*). This suggests that the misclassification likely reflects meningiomas originating from different leptomeningeal layers.

### KYCG detects technical confounders in single-cell and EWAS datasets.

**Fig. 6.**
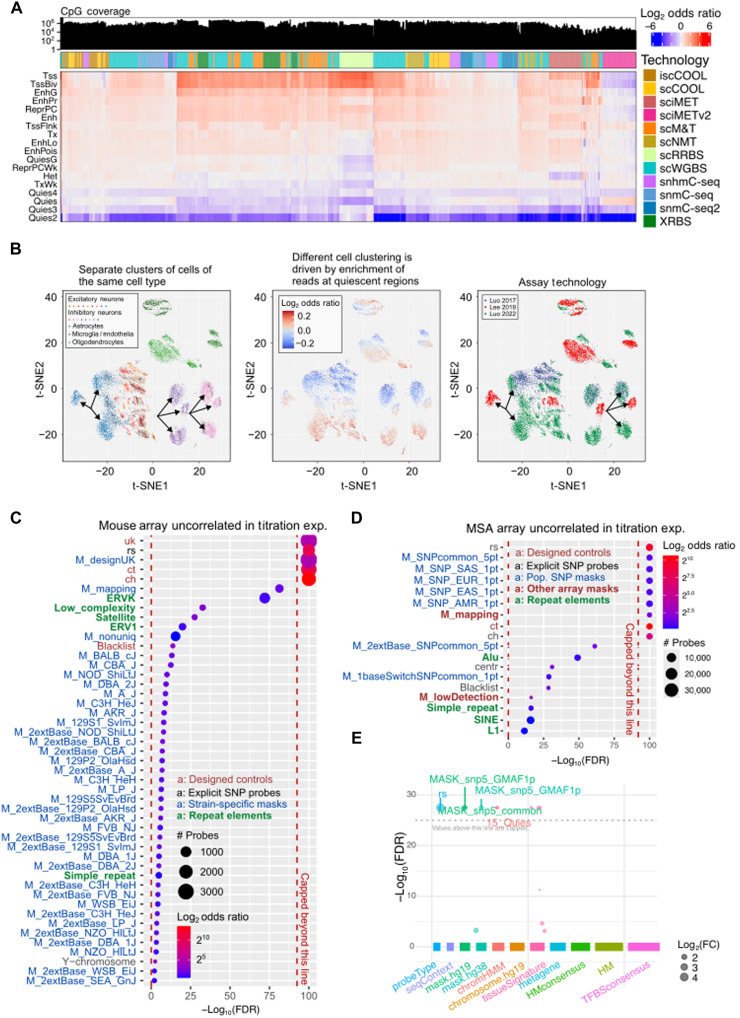
Technical confounder discovery with KYCG in single-cell and EWAS datasets. (**A**) Coverage biases in single-cell methylome technologies of 12 single-cell methylation studies using eight assay technologies. (**B**) Technical variations in three single-cell brain methylation datasets. t-SNE plots illustrate clustering by assay technology and differential capture of chromatin features. (**C** and **D**) Identification and enrichment of probes with poor correlation to methylation titration in the mouse array (C) and the human methylation screening array (D). exp., experiment; Pop., population. (**E**) Detection of ancestry-associated artifacts in EWAS datasets. *P* values are based on Fisher’s exact tests before FDR correction. FC, fold change.

Hidden technical confounders mislead methylation biology interpretation (*8*, *98*) and can be hard to detect even for experienced researchers. The KYCG knowledgebases include CpG sets linked to sequencing- and array-specific artifacts, e.g., methylation measurements influenced by genetic variations or poor coverage uniformity, to enable automatic sanity checks (Fig. 1C). To demonstrate this utility, we first applied KYCG to analyze 12 single-cell methylation studies on mouse tissues using eight assay technologies (Fig. 6A). Clustering these single-cell methylomes by their genomic feature enrichments revealed the impact of profiling technology on coverage uniformity (Fig. 6A). Most single-cell methylome datasets are biased in coverage toward CpG-dense regions, e.g., the transcription start sites (Tss/TssBiv), and depleted in Het and Quies regions, although most library preparation protocols do not intentionally enrich specific genomic regions. As a positive control, this bias is most prominent in single-cell reduced-representation bisulfite sequencing (scRRBS) and single-cell extended representation bisulfite sequencing (scXRBS), as they explicitly target CpG-dense regulatory regions (*99*). iscCOOL (*100*), scCOOL (*101*), and sciMETv2 (*102*) showed a reverse depletion pattern in CpG-rich regions and slight enrichment in Het (Fig. 6A). This reversed nonuniformity was potentially linked to adopting a tailing and ligation method as opposed to the usual postbisulfite adaptor tagging (*100*). Technologies based on the isolated nuclei (e.g., snmC-seq) are depleted in mitochondrial CpGs, while those that profile total cellular DNA are enriched in the mitochondrial genome, reflecting their high copy number (fig. S6A). We integrated two single-cell brain datasets profiled using two different assay technologies. We found that cells of the same cell type form different clusters. KYCG revealed that the difference is primarily linked to the bias in capturing different chromatin features, with Luo *et al.* (*50*) better capturing the Quies regions (Fig. 6B) and being slightly more depleted in TssA/TssFlnk chromatin states, particularly in neurons and oligodendrocytes, compared to the sites covered in Lee *et al.* (*121*) (fig. S6, B and C).

Genetic polymorphism and sequence mappability can substantially affect methylation array measurement but are often overlooked. To demonstrate KYCG’s utility in detecting such artifacts, we used a methylation titration dataset to identify probes whose methylation readings are uncorrelated with the known titrated methylation fractions. KYCG found an enrichment of these probes in probes with known sequence mismatches, single-nucleotide polymorphism (SNP) probes, non-CpG methylation probes, negative control probes, and probes with suboptimal or nonunique mappings (e.g., targeting repetitive elements; Fig. 6, C and D). These enrichments suggested that probe sequence artifacts contributed to the probes’ poor performance, as revealed by the titration experiments. Furthermore, we checked array probes with variable signal intensity in a dataset of nonmalignant human tissues. KYCG identified the enrichment of such probes with mapping and color channel artifacts, suggesting an immediate consequence of probe hybridization and base extension (fig. S6D). Last, we applied KYCG to check CpG sets supposedly associated with ancestry, as in a previous study (*104*). We observed that these CpGs are significantly enriched in methylation readings influenced by human genetic polymorphisms (Fig. 6E), highlighting the critical need to distinguish true methylation quantitative trait loci (meQTLs) or ancestry-linked DNA methylation from measurement artifacts. Our experiment demonstrated the utility of KYCG in conveniently detecting technical confounders in sanity-checking EWAS discoveries.

## DISCUSSION

Efficient enrichment testing tools are critical to the effective learning of omics datasets. CpG sites are the base units for DNA methylation data with a fixed length of 2 base pairs (bp) and a globally depleted prevalence, presenting an intrinsic sparsity. Gene-centric and DMR-based methods, originally designed for other omics data types (*13*, *14*, *105*), may be insufficient at fully capturing methylation biology. Gene-centric methods suffer from a CpG-gene linkage challenge and do not cover intergenic changes, which are now also known to have a regulatory role. On the other hand, DMR-based approaches assume that the methylations of nearby CpGs vary at a certain genomic scale, are coregulated by common chromatin features, and should be analyzed as units. However, this assumption can break down when methylation biology functions at finer or broader genomic scales. For example, TFBS often span just 5 to 30 nucleotides and may involve only single CpGs. In such scenarios, a base-level approach, as in KYCG, can be more sensitive at capturing fine-scale patterns. KYCG benefits not only sparse but also nonsparse datasets in providing multiscale interpretations of discrete methylation datasets.

Furthermore, many population-scale epigenetic studies operate within a “CpG subspace,” such as that set by Infinium microarray design. CpG-indexed enrichment analysis is well suited for these contexts, as implemented by existing tools (*31*, *32*). However, a unified framework that generalizes across data types—including sequencing-based assays that may (e.g., WGBS) or may not (e.g., RRBS) target a fixed CpG set—has been lacking. Toward this goal, we conducted in silico experiments to evaluate the stability of enrichment testing across different CpG subspaces. Our analysis suggests that, when the proper testing universe is used, enrichment results from array-defined CpG subspaces faithfully track results from whole-genome datasets, except in extremely sparse scenarios (Fig. 1E). The results likely depend on the query and knowledgebase sets. Using CTCF binding sites as the query, we observed a slight reduction in the number of significant terms relative to uniformly downsampled data of similar genome coverage (Fig. 1E). This is likely due to array-based spaces being biased toward genic and enhancer regions, which may miss intergenic signals. Nonetheless, the top enriched terms remain stable. As methylation microarrays have much smaller CG subspaces, this resilience of enrichment to sparsity would justify the adoption of an array technology for lower experimental and computational costs.

A key strength of KYCG is its unified design that integrates data with curated resources, agnostic to assay platforms. For common array platforms (*67*, *106*–*110*), KYCG precomputed knowledgebases indexed by CpG probe IDs (“cg” numbers). For sequencing data–based knowledgebases, KYCG dynamically sets the appropriate background universe based on the query scope. This flexibility enables consistent enrichment analysis across both array and sequencing platforms, facilitating integration of data with knowledge derived from diverse assay types.

Beyond defined CpG subspaces, KYCG scales base-level interpretation to highly sparse DNA methylome datasets, including single-cell (e.g., snmC-seq or sci-MET) and spatial methylomes (e.g., Spatial-DMT) (*46*). These assays offer high-resolution insights but suffer from signal dropout and low per-site coverage, limiting traditional DMR-based approaches. Aggregation to pseudobulks can also be challenging when not enough cells of the cell type are captured. KYCG offers a solution for studying “dirty” differential methylations where the difference per locus is not statistically examined and DMR boundaries are murky. This strategy may also benefit biological scenarios of global but subtle methylation changes, e.g., methylation reader defects (*111*).

Key to the feasibility of comprehensive testing is the efficiency of KYCG in scanning the whole genome. Compared to gene enrichments focusing on ~30,000 human genes, enumerating ~28 million CpGs imposes a major computational hurdle to CG-based enrichment testing. When the knowledgebases are small and CGs can be indexed in the CG subspaces, one could adopt the traditional approach of set comparisons. However, a more efficient approach is needed when the queries and knowledgebases become larger. Here, we explored both pathways for addressing this hurdle and provided flexible computational solutions. We index the CGs based on the genomic coordinates for large queries and knowledgebases and use a vectorized counting approach to calculate the set overlaps quickly. This substantially enhanced the performance of set comparisons and enabled the efficient testing of thousands of knowledgebases. The same idea can apply to 5hmCs and non-CpG methylations, which are greater in number and more memory demanding. More powerful compression methods may be used to further enhance computational efficiency.

In implementing KYCG’s strategy, we noted that CpG-indexed enrichment testing requires both query and knowledgebase sets, and potentially the universe sets, to share the same CpG index. This is likely the reason some tools such as HOMER natively do not support 2-bp queries. While tools such as LOLA (*17*) can accept 2-bp queries, bias arises if the knowledgebases remain interval based. Converting these intervals to 2-bp resolution eliminates the bias but greatly increases storage and computation time without efficient indexing, limiting scalability to large numbers of databases. For example, comparing the end-to-end run time of KYCG and LOLA in performing the analysis described in Fig. 2B, KYCG was substantially more efficient (fig. S6E), although the two tools produced similar results.

Automated sanity checks against colinear biology and technical confounders, which may contribute to observed trait associations, are a pressing need even for seasoned scientists. For example, copy number polymorphism has masqueraded as epigenetic silencing events (*98*). Leukocyte contamination may confound the discovery of cancer-associated epigenetic silencing (*112*). Global methylation variation linked to proliferation and impaired DNMT recruitment can be misinterpreted as altered epigenetic aging (*25*, *113*). CpG-rich genomic features, e.g., CpG islands, canyons, and nadirs, overlap extensively and share similar methylation biology, such as mitotic hypermethylation. KYCG represents a step toward addressing this challenge and allows one to check these collinear associations by automatically testing genomic colocalization and comparing enrichment levels. For example, our analysis demonstrated that one can dissect the cell-type context by comparing enrichment levels of the same histone modification features but in different cell types. We also cautioned array-based meQTL discovery due to SNP-originated reading artifacts (*22*). Further expanding and improving the comprehensive collection of knowledgebases is critical to keeping awareness of all hidden biological and technical links.

## MATERIALS AND METHODS

### Whole-genome encoding and compression via YAME

The KYCG framework is designed to streamline whole-genome–wide CpG encoding, data storage, and statistical analyses, leveraging efficient compression and parsing capabilities provided by its core component, YAME (Yet Another MEthylation analysis tool). To minimize storage requirements, genomic coordinates are not explicitly stored. Instead, all knowledgebases and query datasets are preprocessed and indexed according to a default ordering of CpGs based on the reference genome (e.g., GRCh38), with or without contig information. The genomic coordinates are compactly stored separately and can be flexibly combined or built using generic tools such as AWK and BEDTools (*35*). This coordinate-free design reduces redundancy while ensuring consistency across datasets.

YAME, the command-line tool within KYCG, handles the encoding, parsing, and compression of CpG-related data. A combination of bit-packing, Run-length encoding, and the DEFLATE algorithm is used for sparse methylomes dominated by zeros, substantially reducing file sizes for optimal storage, inflation, and access. Categorical data, such as sequence context or chromosome annotations, are compressed using a specialized state encoding scheme. This separates textual state definitions from indices, optimizing repetitive patterns for space savings. For methyl-seq data, YAME uses a unique MU specification, where methylated (M) and unmethylated (U) read counts are stored in a 64-bit integer. The upper 32 bits represent the methylated allele (M), and the lower 32 bits represent the unmethylated allele (U). This encoding is both space efficient and computationally optimized. These integers are further compressed, ensuring compact storage for large datasets.

YAME also enables flexible data manipulation. It supports combining multiple knowledgebases or datasets into a single indexed file, enabling random-access queries with constant time complexity. For extending datasets to higher dimensions (e.g., non-CpG methylation signals or larger genomes), YAME supports data inflation to different levels of precision. This feature allows it to function efficiently in memory-constrained environments. Furthermore, YAME provides extensive functionality for data manipulation, including efficient subsetting of sites and samples, aggregation, masking, downsampling, chunking, and performing rowwise operations. These features make YAME a versatile tool for preparing and analyzing complex datasets.

### Comprehensive CpG annotation

Multilayer CpG annotations are organized as knowledgebases, encompassing 12,114,567 CpG-indexed datasets systematically curated for automated discovery and analysis (see Data and materials availability). This annotation integrates data from human and mouse genome sequences, annotations, and extensive public resources, including 11,806 bulk and 480,012 single-cell sequencing and array-based profiling studies, as well as EWAS projects (table S1A). The annotations are organized into four broad categories of testing domains: (i) sequence features: includes *k*-mers, tetranucleotides, and TF binding motifs; (ii) genomic features: includes chromatin states, histone modifications, gene associations, local modules of CpGs correlated in methylation levels across tissues, transposable elements, TFBS, and evolutionary conservation; (iii) trait associations: includes cell-type–specific methylation, human EWAS associations, and epigenetic clocks; and (iv) technical associates: includes sequence maskers, array hybridization artifacts, and extension masks. Each testing domain includes a varying number of CpG sets linked to biological and technical ontologies.

Sequence features: This category includes key sequence composition metrics such as CpG density, GC fraction, sequence motifs, and *k*-mer contexts. Transcription factor binding models were obtained from HOCOMOCO (*114*), and motif locations in the human and mouse genomes were identified using the FIMO tool from the MEME suite (*115*). These motif locations were extended by ±10 bp to define corresponding CpG sets. Tetranucleotide sequence contexts were integrated with three-dimensional (3D) chromatin compartment data to capture CpG sets associated with biologically relevant features, such as PMD solo-WCGW sequences, which are indicative of replicative methylation loss (*25*), and other sequence contexts known to be more subject to biased DNMT (*116*) or TET-mediated modifications (*117*). For most sequence feature knowledgebases, including tetranucleotide contexts, CpG references were standardized by merging the C and its complementary palindromic G. In addition, stranded CpG sets were constructed to assess strand-specific preferences for hemimethylation and non-CpG methylation, providing deeper insights into sequence-context–specific methylation patterns.

Genomic features: CpG sets were characterized across genomic scales, from large-scale features such as Hi-C AB compartments and topologically associating domain (TAD) domains to smaller-scale events such as histone modifications and TFBS. ChromHMM annotations, TFBS, and histone modifications were used to construct both consensus and cell-type–specific knowledgebases. Data were sourced from Cistrome (*118*) and ReMap 2022 (*103*), which integrate ENCODE data. The peaks were intersected with human and mouse reference genome CpG coordinates. The top 50,000 to 100,000 CpGs with the highest overlap frequencies (including variations due to ties) were selected to construct consensus TFBS and histone modification knowledgebases. Different consensus ChromHMM annotations were taken from the human and mouse data generated in the Roadmap Epigenomics Mapping Consortium (*119*) and ENCODE (*24*, *120*), targeting primary tissue and cell lines, respectively. To address the underrepresentation of cell-type– or tissue-specific chromatin states (e.g., enhancers or promoters) in consensus annotations, full-stack ChromHMM segmentation (*77*, *81*) was incorporated to construct CpG-indexed knowledgebases for specific cell or tissue types. These were refined into MU-style knowledgebase sets by calculating the frequency of CpG overlaps across samples to capture consensus and specific features. Additional features include the integration of the PhastCons evolutionary conservation score to capture conservation metrics and indexing metagene data relative to gene coordinates for positional annotations of CpGs within genes. Gene links were derived for CpGs within a region from 10-kb upstream TSS to transcription termination sites. Enhancer-overlapping subsets were constructed on the basis of CpGs in regions marked by H3K4me1 and H3K27ac and the absence of H3K4me3, defining active enhancers. These annotations enable quick data summaries, such as using metagene knowledgebases for generating metagene plots and flanking sequence sets for sequence logo visualizations, ensuring comprehensive and flexible genomic analyses.

Trait associations: This category includes cell-type–specific methylation as identified by single-cell and sorted cell methylome profiles and those linked to human traits, as primarily identified from previous array experiments. To construct cell-specific CpG knowledgebase sets, BED/bigWig files for single-cell brain (*50*, *78*, *121*), sorted pan tissue (*79*), and sorted immune cell WGBS (*122*) data were downloaded and used for marker identification. To reduce the sparsity of single-cell brain data, pseudobulk methylomes were generated by averaging methylation over the cell-type labels obtained by previously reported unsupervised clustering analysis. To define cell signatures, we first developed 1038 contrast groups (table S2) by manually curating the hierarchy of cell types, each defining a sample set. The curation was guided by global methylome similarity and biological knowledge (Fig. 4A). We then investigated every pair of sample sets across major cell-type groups and hierarchically within major groups. Targeting these contrast groups, we performed a nonparametric discriminant analysis as follows: Pairwise Wilcoxon rank sum testing was performed between the target and the background groups at each CpG site to identify cell-specific markers. CpG sites with an area under the curve (AUC) > 0.95 and a difference in β value of >0.5 between the target and the background groups were selected. Cell signature knowledgebases were tested for enrichment against consensus and full-stack ChromHMM knowledgebases in KYCG using the testEnrichment function. For human trait associations, 1067 EWAS studies were curated from the literature and EWAS databases [EWAS catalog (*123*) and EWAS atlas (*124*)] and converted to knowledgebases by intersecting the trait-associated CpG probes with each array platform.

Technical associates: This category includes CpG groups useful for controlling data quality in sequencing and array experiments. Besides checking for sex and mitochondrial chromosome enrichment, sequence-based knowledgebases include the ENCODE exclusion list (*125*), centromeres, telomeres, and micro- and macrosatellite sequences. Probe array masks were obtained from previous studies (*9*). Briefly, they cover probe hybridization and extension artifacts due to sequence polymorphism and nonuniqueness.

### Knowledgebase cross-validation

The curated CpG knowledgebases are diverse in biological category and size (fig. S1B). To understand the redundancies and relationships between the knowledgebase sets, we computed the NPMI, a statistical measure of co-occurrence (−1 = never, 0 = independence, and 1 = always co-occurs) for each pair. Figure S1C shows a graph of a small subset of intergroup knowledgebase sets sharing the highest NPMIs (>0.5) across all computed pairs. The remaining edge list was graphed in Cytoscape (*126*) version 3.9.1 with the Prefuse Force Directed layout. NPMIs between histone modifications were graphed in ComplexHeatmap (*127*) version 2.19.0. Although it was not uncommon for knowledgebases from different groups to share some CpG sites after thresholding for NPMI, five general communities emerged: (i) CpG islands and TSS, (ii) gene bodies, (iii) Het regions, (iv) bivalent and polycomb repressive complex 2 (PRC2) targets, (v) CTCF binding sites, and (vi) enhancer-like elements. NPMI was also computed for every cell-signature knowledgebases. Sets with an NPMI >0.4 were selected for visualization using the Circlize package (version 0.4.15) (*128*).

We explored the overlap of the 83 histone modifications and 1188 TFBS knowledgebases with ChromHMM genomic features. Related histone modification–overlapping CpG sets are clustered together based on NPMI, forming distinct groups (fig. S1D). Notably, the promoter group is overrepresented by various activating histone acetylation and H3K4me3 marks. Other histone modification–overlapping CpG knowledgebases are organized into broad categories representing bivalent chromatin, gene transcription, and Het (fig. S1D). Transcription factor binding sites rarely co-occurred with Het and Quies regions, with mean NPMIs of −0.244 and −0.236, respectively. A total of 161 TFs of the 1188 (13.6%) did not have an NPMI > 0.25 with any ChromHMM feature. This group of TFs was enriched in the ZNF family of proteins (*P* = 9.952 × 10^–10^; Fisher’s exact test), and gene ontology analysis revealed enrichment relating to DNA replication. Of the remaining TFs, 944 (79%) showed the highest NPMI with TssA, consistent with TFs generally binding adjacent to promoters. A total of 31 (3%) TFs displayed the highest preference for EnhA1 regions, 21 (2%) for TssBiv regions, 13 (1%) for genic enhancers (EnhG2), 10 (0.8%) for TssFlnkU, 4 for Tx, and 3 for ZNF_OR_Rpts. Overall, TFs are generally localized with Tss elements and enhancers (fig. S1E). TFBS-overlapping CpGs were analyzed across multiple experiments, aggregating overlaps to compute NPMI with ChromHMM features. TFBS with NPMI > 0.25 were grouped by their highest NPMI ChromHMM feature. Gene Ontology analysis for TFs in each group was performed using Enrichr (*129*). This validates the construction and confirms the expected biological relationships among the knowledgebases.

To validate cell-type–specific signatures, each knowledgebase was first tested for enrichment in gene knowledgebases within 10 kb of the query CpGs, identified with the buildGeneDBs function. Enriched genes [false discovery rate (FDR) < 0.05; Fisher’s exact test] for each signature branch were overlapped with the marker genes for each nontumor human cell type from the CellMarker2.0 database (*130*), and cell types from pairs that had four or more overlapping genes were selected for visualization in ComplexHeatmap (version 2.19.0) (*127*). For brain cell enrichment testing, one versus all signatures for excitatory neurons, inhibitory neurons, and glia were tested for enrichment against gene (identified with buildGeneDBs), consensus ChromHMM, and TFBS knowledgebases.

### CpG set enrichment testing

Building on YAME’s ability to rapidly compute CpG counts and overlaps (with an optional universe set constraint), the KYCG R/Bioconductor package provides statistical analysis functionalities and visualization for enrichment results. For pairwise methylome analysis, the YAME’s pairwise function efficiently identifies differential methylation CpG sets (DMCs) that represent various contrasts (e.g., hypermethylation, hypomethylation, or both combined) with customizable filters, using the set of CpGs involved in the comparison (covered in both profiles and comparable) as the universe. KYCG uses the hypergeometric distribution as the null hypothesis for enrichment testing. The package supports fast calculation of Fisher’s exact test statistics (via R’s phyper function) and FDR correction, offering one- and two-sided testing options. While efficient, this test assumes statistical independence among CpGs. Multiple test corrections, by default via Benjamini-Hochberg, are done within each testing domain to avoid domain size imbalance. This is justified by the distinct hypothesis space with different term counts, biological relevance, and structural organization.

In addition, KYCG uses a gene set enrichment analysis–like strategy to compare set-based query or knowledgebases and continuous vector variables on a defined universe. Significance is assessed using a Kolmogorov-Smirnov test on the permuted null distribution, with a Gaussian approximation of the null offered as an efficient alternative for large query or knowledgebases. In addition, the framework integrates gene-CpG associations, enabling pathway-level analyses of genes linked to enriched CpGs. A suite of visualization tools, including dot plots, waterfall plots, volcano plots, and track plots, is available to ensure a clear and interpretable presentation of results. Enrichment testing considers a universe set built for each experiment. YAME binarize and YAME pairwise function conveniently produce a paired target and universe set from data for subsequent enrichment testing.

### KYCG performance and stability

For each platform (whole genome, EPIC, and HM450), random queries of 1 million, 0.5 million, and 0.1 million were generated by sampling (with replacement when necessary) the respective platforms’ universe space. The queries were tested for enrichment in consensus ChromHMM features, using the respective platform as the background universe space. Testing for each query size–platform pair was repeated 100 times. Compute times for set-based testing in R were measured using the Sys.time() function. For vectorized testing, the command-line time function was used. Compute times were measured only for the Fisher’s exact testing process and not the time elapsed for I/O of the knowledgebase and universe files or query generation. Memory usage was tested using the same queries and ChromHMM features. Maximum resident set size was recorded with time -f “%M” parameters for the maximum memory usage from the time of loading files to testing enrichment. To compare whole-genome computing of enrichment statistics, BEDTools intersect (v2.30.0) was used to intersect query and knowledgebase sets using the -sorted option, followed by counting in AWK (v5.1.0). For methylation aggregation over knowledgebase sets, BEDTools intersect and groupby functions were used. Enrichment statistics and methylation aggregation in KYCG were both computed using the yame summary -m function.

CTCF binding sites were identified from ENCODE chromatin immunoprecipitation sequencing data (*131*) and intersected with the reference genome (GRCh38) CpG coordinates to use as a query for enrichment testing in ChromHMM features. The GRCh38 reference genome CpG space was uniformly downsampled by factors of 2, 2^2^, 2^4^, 2^6^, 2^8^, 2^10^, 2^12^, and 2^14^ to create universe subsets for enrichment testing. RRBS data from 17 tissues were downloaded from ENCODE (*24*). Fifty iterations of downsampling and enrichment testing were performed for each universe size and type. RRBS and array data were not downsampled.

### Genomic proximity testing

Proximity testing of hyper- and hypomethylated CpG markers was modeled with a Poisson distribution with a λ parameter representing the number of CpGs occurring in fixed 1500-bp intervals. For a given query set of CpGs, a null distribution was generated by performing 1000 simulations of random samples of equal size to the query and calculating the mean number of events (CpGs co-occurring in a 1500-bp interval) as the λ parameter. This λ was used as the Poisson point estimate to compute the probability for the number of co-occurrences in the query set.

### Benchmarking datasets

Nucleosome occupancy and methylome sequencing (NOMe-seq) data from PGCs were downloaded from a prior study (*132*). Methylated CpGs (methylation fraction ≥ 0.3, minimum coverage = 1) were used as a query for enrichment testing against full-stack ChromHMM, histone modification, and TFBS knowledgebase sets using all CpGs with non–not available (NA) values for each sample as the universe. Enrichment testing was performed using the YAME summary function.

Single-cell DNA methylome data from Bian *et al.* (*38*) and Liu *et al.* (*39*) were downloaded and stored using YAME. Fifty pairs of cells were randomly selected, and methylation differences were calculated to define hyper- and hypomethylated sites (methylation differences of 1 or −1). The universe set is defined as sites covered by both cells. Spearman correlation was used to compare the enrichment ordering of the sampled pairs with the most deeply sequenced pairs (i.e., the pair with the greatest number of CpGs covered in both cells). Differential methylation regions were merged from differentially methylated sites within 10-kb windows and used as inputs for HOMER (*13*) motif analysis via findMotifsGenome.pl. For TFBS analysis, single-cell data were downloaded from Luo *et al.* (*50*), and methylation was aggregated over the 1188 TFBS knowledgebase sets using the YAME summary function. Cells were grouped according to the major class label reported by the authors. Wilcoxon rank sum testing was performed between the target cell type and the background groups at each TFBS feature, and each TFBS that discriminated the target cell type with an AUC of 0.8 or higher was selected for further analysis.

Cancer WGBS data were obtained from TCGA. Two cancer types (bladder and breast cancer) were selected. Compared to adjacent normal tissues, hypermethylated sites were tested against cell-type–specific histone modification features. Pseudobulks from spatial embryo E11.5 methylation data were merged for the heart and neural tube regions (3 × 3 pixels), and methylation differences were tested to demonstrate cell-type–specific TFs.

For 5hmC and Oxford Nanopore sequencing analysis, SIMPLE-seq (*133*) and snhmC-seq (*57*) datasets were downloaded and processed into YAME-compatible formats. Pseudobulks were merged for each major brain cell type. Pairwise comparisons of the snhmC-seq data among the four major brain cell types were conducted using the YAME pairwise function, focusing on 40% or more methylation differences. ONT 5mC and 5hmC data (Supplementary Materials) were analyzed against chromatin states across four distinct cell types. ACE-seq (*134*) data from embryonic stem cells were used as a benchmark to validate ONT 5hmC enrichment.

For 5hmC array-based analyses, bACE-array data were obtained from a previous study (*67*). One-versus-all comparisons were performed for the displayed tissue type groups using Wilcoxon rank sum testing between the target and the background group at each CpG site. CpG sites with 5% or more methylation differences and an AUC > 0.8 for discriminating the target tissue type were considered for further analysis. Marker CpGs were linked to genes (GENCODEv19) ±1500 bp from the CpG site. Linked genes for each tissue type were tested for enrichment against the CellMarker 2024 and Human Gene Atlas gene ontology databases using Enrichr (*129*, *135*).

For RNA expression comparisons, cell-type–specific RNA sequencing count data were downloaded from the Human Protein Atlas “RNA single-cell type data” database (*136*), and expression levels of *NKX2-1* were log transformed and plotted across 79 cell types. To evaluate KYCG’s capacity for screening array probe artifacts, we used methylation titrations from prior studies (*34*, *67*, *137*). β values from 10 mouse DNA samples with varying methylation titration levels (0, 5, 10, 25, 50, 75, and 100%) generated by EpigenDx (*137*) were used to test the correlation between beta values and methylation titrations. For each CpG on the MM285 array, Pearson’s correlation was computed between the methylation reading and the expected methylation level of the titration. CpG probes with a correlation coefficient < 0.9 were used as a query to test enrichment in MM285 technical database sets.

### EWAS and predictive model feature interpretation

EWAS trait associations were downloaded from databases (*123*, *124*), and associated CpGs were converted into knowledgebases by intersecting each trait CpG set with array manifests. Each one-versus-rest cell-type–specific methylation knowledgebase was tested for enrichment against the HM450 EWAS trait knowledgebase using the testEnrichment() function, and the top six most significantly enriched traits were plotted for each cell type. Epigenetic clock CpG query sets were downloaded and tested against EWAS trait, gene, cell-type–specific methylation signature, chromHMM, histone modification, and PMD knowledgebases under each clock’s respective assay platform. Gestational aging methylation data were downloaded from Koeck *et al.* (*86*). The Pearson correlation coefficient was computed between the methylation of the clock CpGs in the *HOXB3* gene on the EPIC array and the corresponding sample’s gestational age.

To analyze the central nervous system tumor classifier features, data from Capper *et al.* (*96*) were downloaded and preprocessed using the SeSAMe package (*98*). The 32,000 most variable CpGs were used as features to train a random forest classifier using the randomForest package in R with default parameters. The reference cohort of 2801 samples was used for training, and testing was performed on 1100 samples from the prospective cohort. Importance scores for classifier features were ranked according to the decrease in the Gini index for each CpG. The top and bottom 16,000 CpGs based on the Gini index are considered high- and low-importance features. Differential methylation analysis between correctly and incorrectly classified meningioma samples was performed using the SeSAMe DML() function, and differentially methylated CpGs were tested for enrichment against all knowledgebases. Visualization of the *TNXB* gene was performed using the SeSAMe visualizeGene function.
