# Supplementary material for: KnowYourCG: Facilitating base-level sparse methylome interpretation
Source: Sci Adv. 2025 Oct 24;11(43):eadw3027. doi: 10.1126/sciadv.adw3027 (PMC12551721; doi:10.1126/sciadv.adw3027)
Supplement: Supplementary file 1 — Figs. S1 to S6 Legends for tables S1 and S2 [file sciadv.adw3027_sm.pdf]

Science Advances
AAAS

Supplementary Materials for

KnowYourCG: Facilitating base-level sparse methylome interpretation

David C. Goldberg *et al.*

Corresponding author: Wanding Zhou, wanding.zhou@pennmedicine.upenn.edu

Sci. Adv. **11**, eadw3027 (2025)
DOI: 10.1126/sciadv.adw3027

The PDF file includes:

Figs. S1 to S6
Legends for tables S1 and S2

Other Supplementary Material for this manuscript includes the following:

Tables S1 and S2

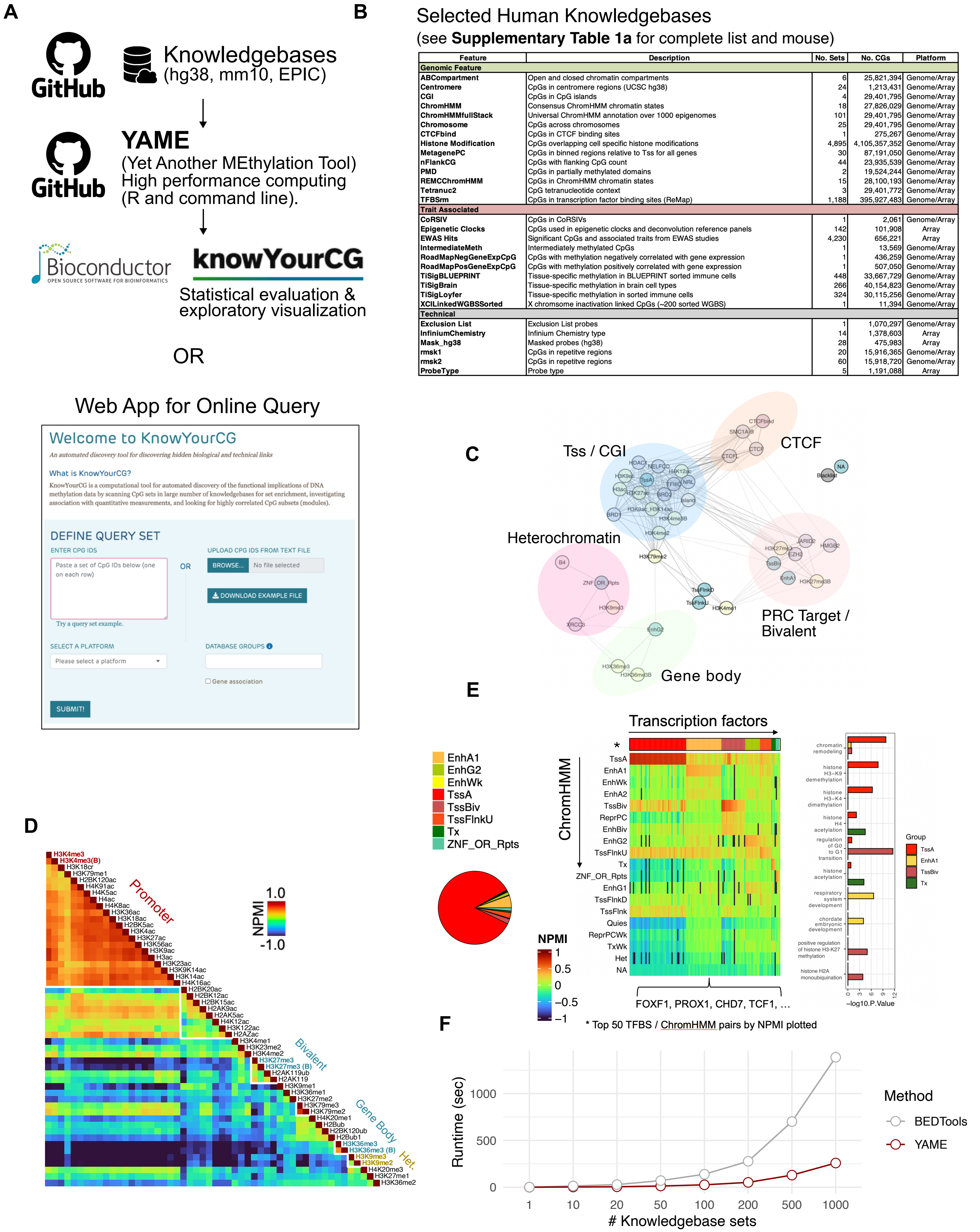

Feature	Description	No. Sets	No. CGs	Platform
**Genomic Feature**				
**ABCompartment**	Open and closed chromatin compartments	6	25,821,394	Genome/Array
**Centromere**	CpGs in centromere regions (UCSC hg38)	24	1,213,431	Genome/Array
**CGI**	CpGs in CpG islands	4	29,401,795	Genome/Array
**ChromHMM**	Consensus ChromHMM chromatin states	18	27,826,029	Genome/Array
**ChromHMMfullStack**	Universal ChromHMM annotation over 1000 epigenomes	101	29,401,795	Genome/Array
**Chromosome**	CpGs across chromosomes	25	29,401,795	Genome/Array
**CTCFbind**	CpGs in CTCF binding sites	1	275,267	Genome/Array
**Histone Modification**	CpGs overlapping cell specific histone modifications	4,895	4,105,357,352	Genome/Array
**MetagenePC**	CpGs in binned regions relative to Tss for all genes	30	87,191,050	Genome/Array
**nFlankCG**	CpGs with flanking CpG count	44	23,935,539	Genome/Array
**PMD**	CpGs in partially methylated domains	2	19,524,244	Genome/Array
**REMCChromHMM**	CpGs in ChromHMM chromatin states	15	28,100,193	Genome/Array
**Tetranuc2**	CpG tetranucleotide context	3	29,401,772	Genome/Array
**TFBSrm**	CpGs in transcription factor binding sites (ReMap)	1,188	395,927,483	Genome/Array
**Trait Associated**				
**CoRSIV**	CpGs in CoRSIVs	1	2,061	Genome/Array
**Epigenetic Clocks**	CpGs used in epigenetic clocks and deconvolution reference panels	142	101,908	Array
**EWAS Hits**	Significant CpGs and associated traits from EWAS studies	4,230	656,221	Array
**IntermediateMeth**	Intermediately methylated CpGs	1	13,569	Genome/Array
**RoadMapNegGeneExpCpG**	CpGs with methylation negatively correlated with gene expression	1	436,259	Genome/Array
**RoadMapPosGeneExpCpG**	CpGs with methylation positively correlated with gene expression	1	507,050	Genome/Array
**TiSigBLUEPRINT**	Tissue-specific methylation in BLUEPRINT sorted immune cells	448	33,667,729	Genome/Array
**TiSigBrain**	Tissue-specific methylation in brain cell types	266	40,154,823	Genome/Array
**TiSigLoyfer**	Tissue-specific methylation in sorted immune cells	324	30,115,256	Genome/Array
**XCILinkedWGBSSorted**	X chromsome inactivation linked CpGs (~200 sorted WGBS)	1	11,394	Genome/Array
**Technical**				
**Exclusion List**	Exclusion List probes	1	1,070,297	Genome/Array
**InfiniumChemistry**	Infinium Chemistry type	14	1,378,603	Array
**Mask_hg38**	Masked probes (hg38)	28	475,983	Array
**rmsk1**	CpGs in repetitve regions	20	15,916,365	Genome/Array
**rmsk2**	CpGs in repetitve regions	60	15,918,720	Genome/Array
**ProbeType**	Probe type	5	1,191,088	Array

Supplementary Fig. S1 | KYCG knowledgebase features and genomic annotations (A) KYCG components: YAME, R API, and a web app (B) Summary table of major KYCG knowledgebase sets. (C) Network diagram illustrating relationships between genomic feature knowledgebase sets (D) Heatmap showing hierarchical clustering of histone modification knowledgebase sets based on their mutual NPMI patterns. (E) Transcription factor binding site enrichments in ChromHMM states with a corresponding pie chart showing the most common chromatin states where binding sites have the highest NPMI. Bar plots indicate the enrichment of ontology terms for transcription factors binding each chromatin state. (F) Compute time for aggregating methylation over input knowledgebase sets for YAME (red) and BEDTools (grey).

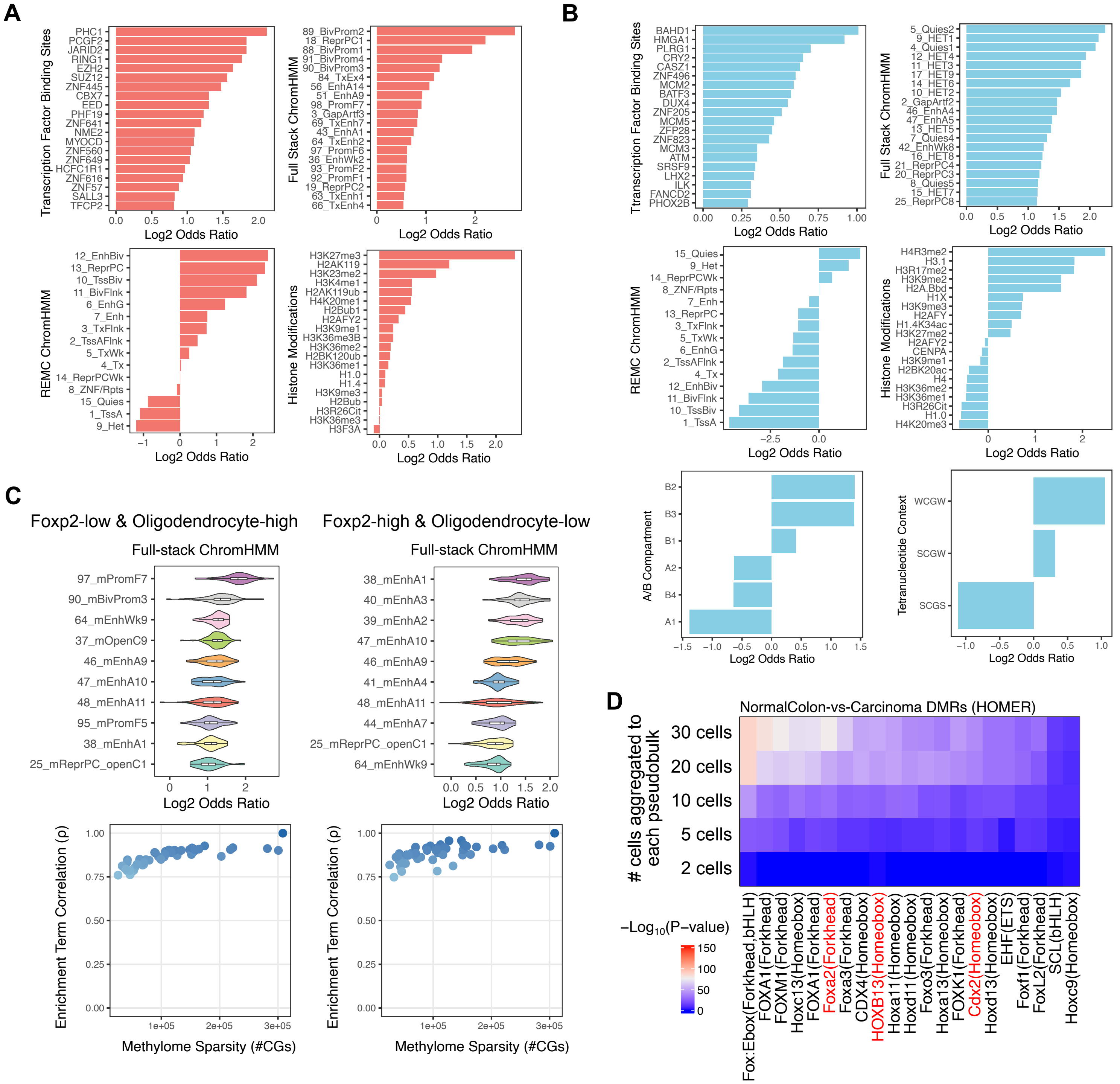

Supplementary Fig. S2 I KYCG genomic feature enrichments for sparse methylomes study (A) Enrichments of transcription factor binding sites, full-stack ChromHMM states, REMC ChromHMM states, and histone modifications for hypermethylated CpG sets derived from a colon cancer cell and an adjacent normal cell. (B) Hypomethylation in colon cancer cell enrichments in quiescent and heterochromatic regions. (C) Comparative analysis of Foxp2 neurons and oligodendrocytes methylation enrichment and correlation consistency across varying levels of methylome sparsity. (D) Testing the enrichment of DMRs called from methylomes of different sparsity levels reveals enrichment of the binding motifs of CDX2, FOX family TFs, and HOX clusters (columns). DMRs were called using DMR-seq. Enrichment of DMR intervals was analysed and statistical significance was evaluated using HOMER. Rows correspond to the number of cells aggregated to the pseudobulks. Each DMR analysis used 2 pseudobulks for normal colon and 2 pseudobulks for cancer.

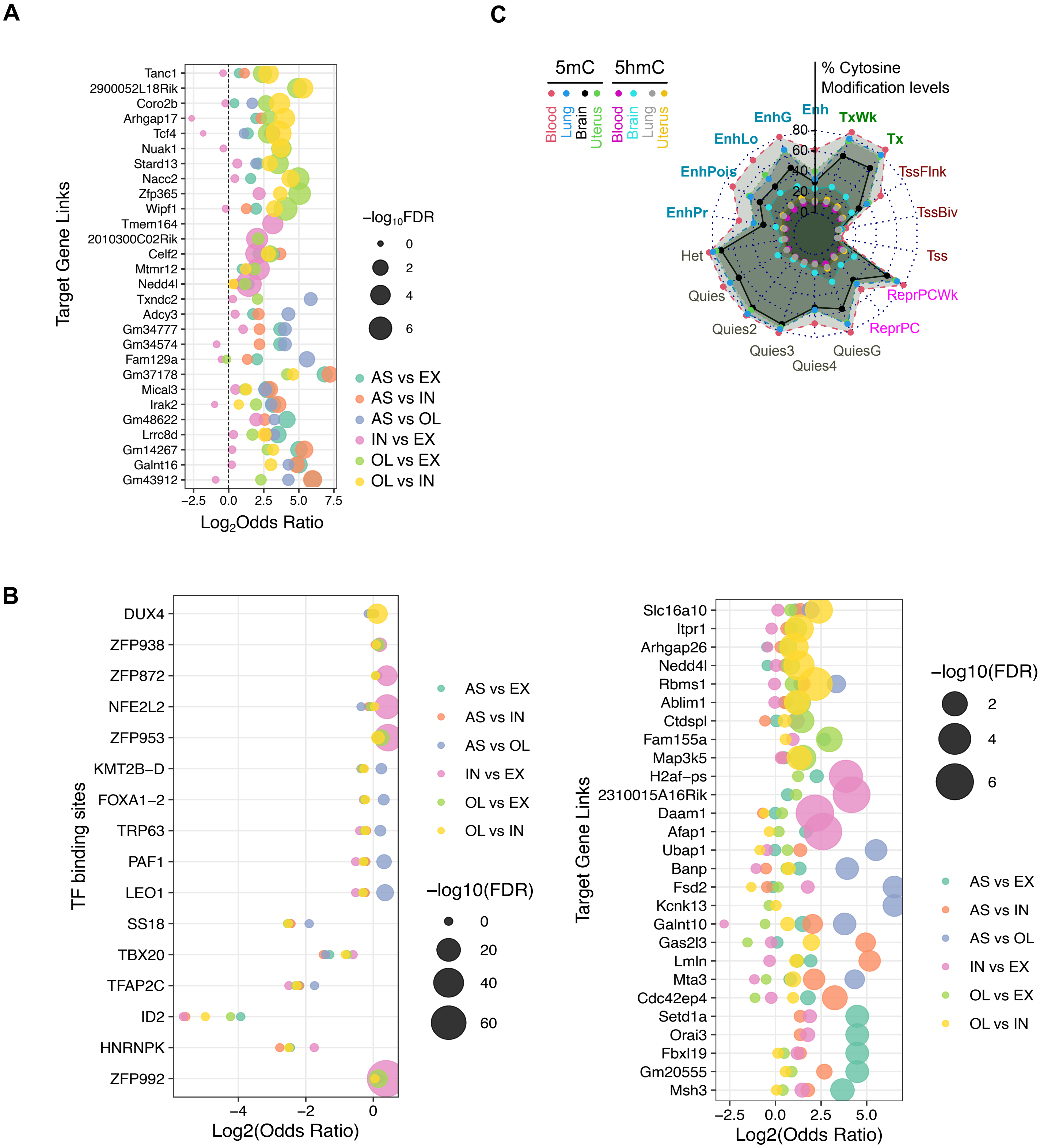

Supplementary Fig. S3 I KYCG analysis of sparse 5hmC and ONT direct detection. (A) Dotplot showing the enrichment in linked genes of CpGs with hyper 5hmC between cell type contrast/comparison groups. Colors indicate cell type comparisons. (B) Dotplot showing the enrichment in transcription factor binding sites (left) and linked genes (right) of CpGs with hypo 5hmC between cell type comparison groups. Colors indicate cell type comparisons. (C) Radar plot showing % 5mC and 5hmC across consensus ChromHMM chromatin states for ONT profiled tissues.

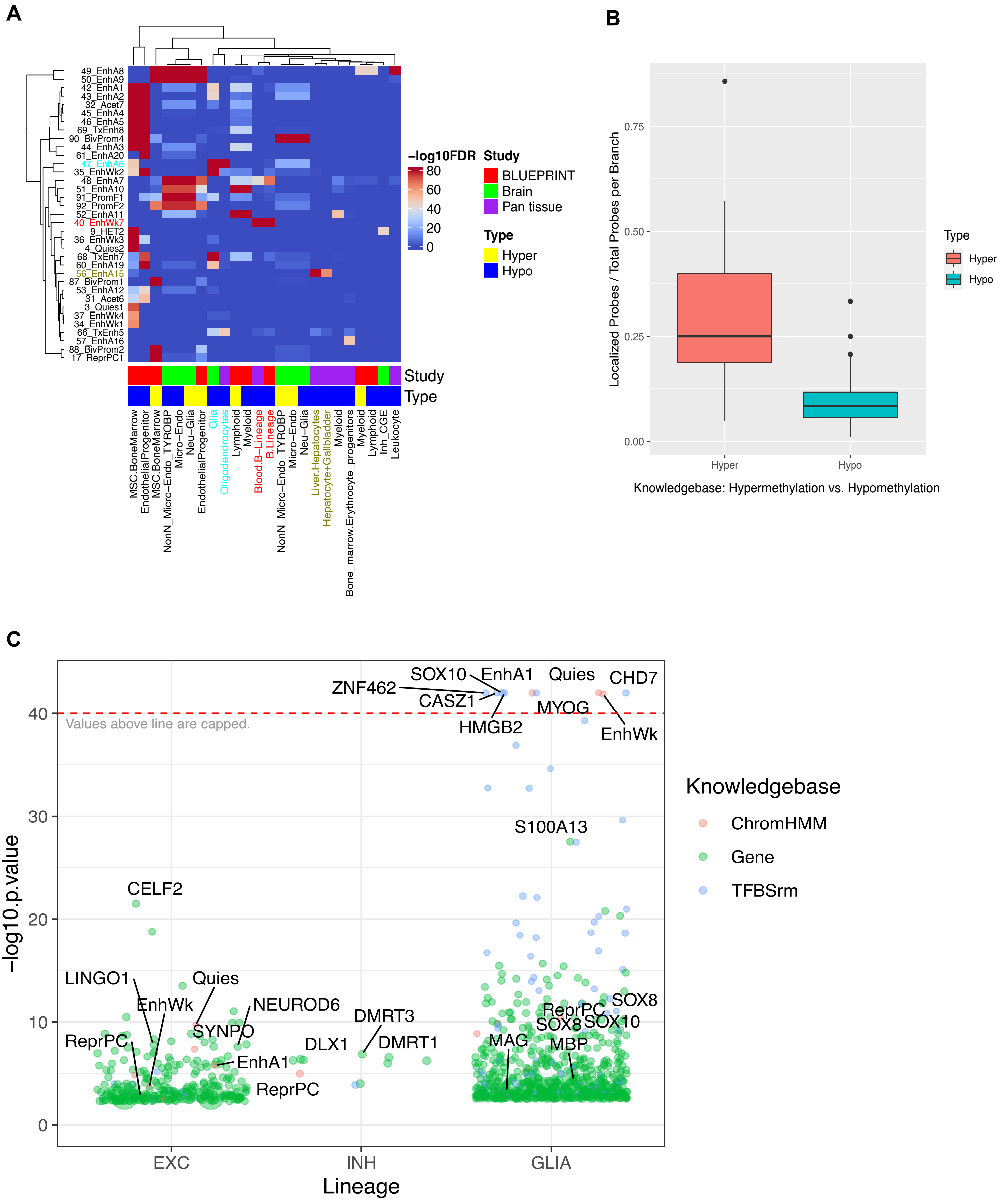

Supplementary Fig. S4 | Enrichment patterns between hyper and hypomethylated CpG signatures across lineages (A) Heatmap and hierarchical clustering for enrichment of hyper and hypomethylated CpG signatures in full-stack ChromHMM states. (B) Boxplot comparing localized probe density for hyper versus hypomethylated cell-type signature knowledgebase types. (C) Dotplot showing enrichment of lineage-specific CpG signatures for major CNS cell types in ChromHMM states, gene, and transcription factor binding site knowledgebase sets.

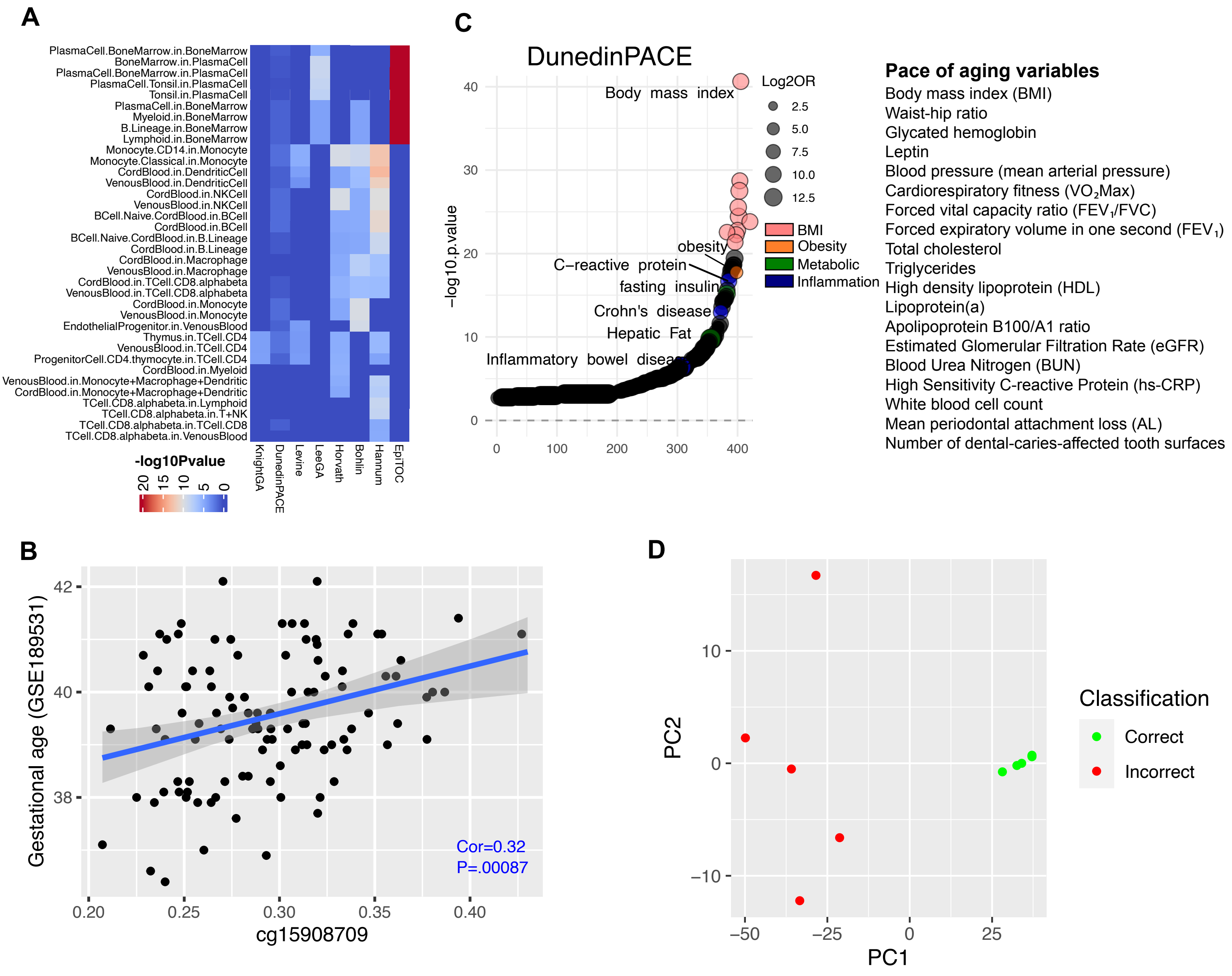

Supplementary Fig. S5 | Functional characterization of predictive model features using KYCG. (A) Heatmap showing enrichment patterns of CpG features across cell types and tissues in epigenetic clock models. (B) Correlation plot between gestational age and methylation at cg15908709 in the HOXB3 locus. (C) Enrichment of DunedinPACE clock features in metabolic and inflammatory EWAS traits. (D) Principal component analysis separates correctly classified and misclassified meningioma samples by methylation features.

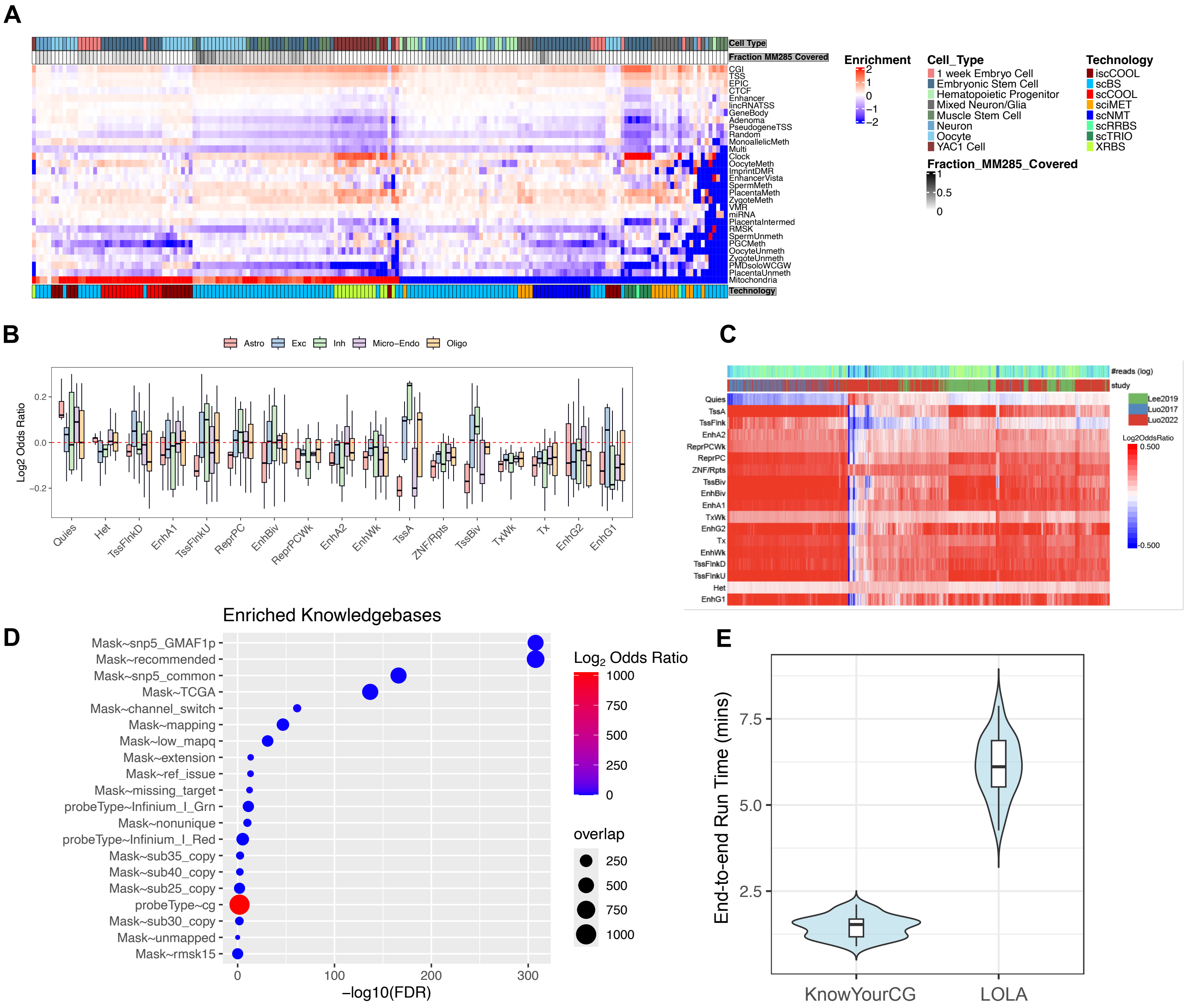

Supplementary Fig. S6 | Detection of technical confounders using KYCG. (A) Heatmap showing enrichment and depletion patterns of genomic features across 12 single-cell methylation studies and eight assay technologies restricted to the MM285 universe space. (B) Boxplot of log2 odds ratios highlighting assay-specific biases between Luo2022 and Lee2019 across five cell types. (C) Heatmap showing enrichment of CpGs profiled in 3 different single-cell brain data sets across ChromHMM features, highlighting assay-specific biases. (D) Dot plot showing the enrichment of CpGs with variable signal intensity in normal human tissues, highlighting confounding by mapping and color channel artifacts. (E) Comparison of run time between KYCG and LOLA using a 2-bp query (as described in Figure 2B). 50 single-cell pairs from colon tumor and adjacent mucosa were analyzed for enrichment of chromatin states relative to transcription factor binding sites. The end-to-end running time, including data input/output, is reported in minutes.

Supplementary Excel Table S1A: Curated KnowYourCG Knowledgebases
Supplementary Excel Table S1B: EWAS studies analyzed for tissue context (as reported in Fig 4H)
Supplementary Excel Table S2: Tissue-specific CpG counts and contrast groups
